# Cytotoxic and Anti-Inflammatory Evaluation of Thai Medicinal Plants from Kampramong’s Anticancer Remedy Against Upper Respiratory Tract Malignancies

**DOI:** 10.3390/ijms27167239

**Published:** 2026-08-13

**Authors:** Nanatphong Paje, Arunporn Itharat, Waipoj Chanvimalueng, Vilailak Tiyao, Weerachai Pipatrattanaseree, Sunita Makchuchit, Pranporn Kuropakornpong, Neal M. Davies

**Affiliations:** 1 Graduate School, Faculty of Medicine, Thammasat University, Klong Luang 12120, Pathum Thani, Thailand; nanatphongpaje@hotmail.com; 2 Department of Applied Thai Traditional Medicine, Faculty of Medicine, Thammasat University, Klong Luang 12120, Pathum Thani, Thailand; 3 Center of Excellence in Applied Thai Traditional Medicine Research (CEATMR), Thammasat University, Klong Luang 12120, Pathum Thani, Thailand; meyamesaya@gmail.com (V.T.); msunita_359@hotmail.com (S.M.); pranporn.kur@gmail.com (P.K.); 4 Department of Otolaryngology, Faculty of Medicine, Thammasat University, Klong Luang 12120, Pathum Thani, Thailand; drwaipoj@gmail.com; 5 Regional Medical Sciences Center 12 Songkhla, Ministry of Public Health, Mueang Songkhla 90100, Songkhla, Thailand; weerachai.dmsc@gmail.com; 6 Katz Group-Rexall Centre for Pharmacy and Health Research, Faculty of Pharmacy and Pharmaceutical Sciences, University of Alberta, Edmonton, AB T6G 2E1, Canada; ndavies@ualberta.ca

**Keywords:** *Caesalpinia sappan*, Kampramong’s anticancer remedies, field cancerization, upper respiratory malignancy, molecular docking

## Abstract

Field cancerization in the upper respiratory tract (URT) heavily drives cancer recurrence. The present study evaluated Thai ethnobotanical ingredients from Kampramong’s anticancer remedies as alternative agents for URT cancer control. Thirty-one ethanolic extracts were screened for cytotoxicity against six URT malignant cell lines (HK1, KB, HONE-1, NPC38, NPC43, and HEp2) and two non-cancerous controls (NP460 and HaCaT) using the sulforhodamine B (SRB) assay. The selectivity index or IC_50_ ratio of normal cells/cancer cells was also determined for selective cytotoxicity of extracts and compounds. Anti-inflammatory activity was measured using the nitric oxide (NO) reduction assay. Antioxidant capacity was measured using the DPPH radical scavenging assay. Total flavonoid content (TFC) was measured due to the main risk reduction factor. Multivariate analyses (Hierarchical Cluster Analysis; HCA and Principal Component Analysis; PCA) were used for the relationship of flavonoids, NO inhibition drive to cytotoxicity. The best plant extracts and their cytotoxic compounds were also predicted to have their target molecular mechanisms by molecular docking. HCA identified *Caesalpinia sappan*, *Coscinium fenestratum*, and *Tectona grandis* as exhibiting potent cytotoxicity against all malignant cells, with *C. sappan* being the most potent. PCA revealed that flavonoid content and nitric oxide inhibition were the critical factors driving cytotoxicity (factor load 71.93%). Molecular docking of *C. sappan* compounds, brazilin and sappanchalcone, showed high binding energies with multiple core target proteins (MAPK1, MAPK3, SRC, AKT1, and GRB2), indicating a synergistic multi-compound, multi-target mechanism, representing a hypothesis for future mechanistic validation. Kampramong’s ethnobotanical remedies, particularly *C. sappan*, are promising candidates for URT malignancy treatment and field cancerization prevention, offering valuable data for future drug development.

## 1. Introduction

Cancer is the leading cause of death in Thailand. Head and neck cancer (HNC) is the sixth most common cancer worldwide and is one of the top five cancers in Thailand; the leading HNC in men are oral, nasopharyngeal, and laryngeal carcinomas, while in women, they are thyroid and oral cavity carcinomas [1].

Locoregional recurrence, metastasis, and second primary cancer are generally considered the major causes of treatment failure in HNC. Field cancerization is generally accepted as the rationale for the higher incidence of subsequent cancer formation than recurrences or additional primary cancer [2]. The same anatomical mucosa makes areas susceptible to malignant formation when continuously exposed to carcinogens or free radicals. A second primary tumor in HNC occurs in 10% to 40% of cases [3]; as a result, patients with nasopharyngeal cancer develop secondary cancers in the oral cavity or other pharyngeal and laryngeal areas, according to field cancerization.

Treatment of upper respiratory tract (URT) cancer is based on tumor staging. The primary treatment modality is radiotherapy or chemotherapy. However, most patients suffer from long-term side effects mainly due to conventional therapy. Furthermore, the standard chemotherapeutic agent, cisplatin, also repeatedly leads to drug resistance and numerous adverse outcomes [4].

Epidemiological studies have shown an inverse relationship between flavonoids and the risk of HNC [5]. Meanwhile, some local habits such as chewing betel, smoking, alcohol consumption, and chronic inflammation of the upper respiratory tract are associated with a higher incidence of HNC [4]. On the other hand, antioxidants play an essential role in reducing these risks [6,7].

Traditional Thai Medicine (TTM) is widely known as an alternative cancer treatment. Several Thai ethnobotanical plants have shown anticancer activity against various cancer cell lines. Therefore, many ethnobotanical plants have been studied as alternative therapeutic agents [8]. In Thailand, Abbot practitioner at Kampramong temple, Sakon Nakorn province used Thai traditional remedy to treat cancer patients for more than 20 years. Kampramong’s anticancer remedies consist of thirty-one herbal medicines. These remedies have been associated with reported successful treatment outcomes [9]. The previous report of Kampramong temple showed that the case report of stage 4 nasopharyngeal carcinoma was successfully treated because the NPC patient is still alive nowadays more than 5 years [9] The cell lines of nasopharyngeal carcinoma were justified as a field cancerization model. HK1, HONE 1, NPC38, NPC43 are Epstein-Barr Virus (EBV) positive nasopharyngeal carcinoma representing latent infection driven carcinogenesis. Differentiation status: HK1 (well differentiated), HONE 1 (poorly differentiated), NPC43 (undifferentiated), covering the spectrum observed clinically in HNSCC [10]. KB is HeLa-derived oral SCC and EBV-negative. HEp2 is a laryngeal SCC representing an aerodigestive tract field.

Bioinformatics-guided target prediction and molecular docking have become established complementary tools in ethnopharmacological research, allowing candidate bioactive constituents identified from crude plant extracts to be linked computationally to specific human protein targets before committing to costly downstream mechanistic assays. In the present work, protein targets of relevance to head and neck carcinogenesis (MAPK1, MAPK3, SRC, AKT1, and GRB2) were identified using the STRING and PharmMapper target-prediction databases (Section 4.8), and their prognostic relevance to head and neck squamous cell carcinoma survival was further evaluated using TCGA and GEO clinical datasets (Section 4.9). Extraction conditions for the ethanolic plant extracts, including a seven-day maceration period in 95% ethanol, are detailed in Section 4.2.

It was hypothesised that ethanolic extracts of Kampramong’s anticancer remedies retain selective cytotoxic activity against upper respiratory tract (URT) malignant cell lines relative to non-cancerous controls, and that this activity is mechanistically linked to their flavonoid content and antioxidant and anti-inflammatory potential. To test this hypothesis, the present study had three specific objectives: first, to screen the thirty-one ethanolic extracts of Kampramong’s anticancer remedies for cytotoxicity against a panel of URT cancer cell lines representing a field cancerization model; second, to apply principal component analysis (PCA) and hierarchical cluster analysis (HCA) to the cytotoxicity, antioxidant, anti-inflammatory, and flavonoid content data to identify the biological activity patterns most strongly associated with reduced HNC risk; and third, to subject the most promising plant extract to a molecular docking study against core protein targets relevant to head and neck carcinogenesis, in order to generate a testable hypothesis for its underlying mechanism of action.

## 2. Results

### 2.1. Extraction Yields of Plant Components in Kampramong’s Anticancer Remedies

The botanical details, biological activity, and traditional use of Thai ethnobotany components in Kampramong’s anticancer remedy are shown in Table S1. The percentage yields of all plant ingredient extracts were in the range of 1.01–15.54%. The highest extract yield was from *Bauhinia strychnifolia* (15.54%). All the percentage yields are shown in Table S1.

### 2.2. Cytotoxic Activity of Plant Extracts Against Malignant Cell Lines of the Upper Respiratory Tract by SRB Assay

**Figure 1:**
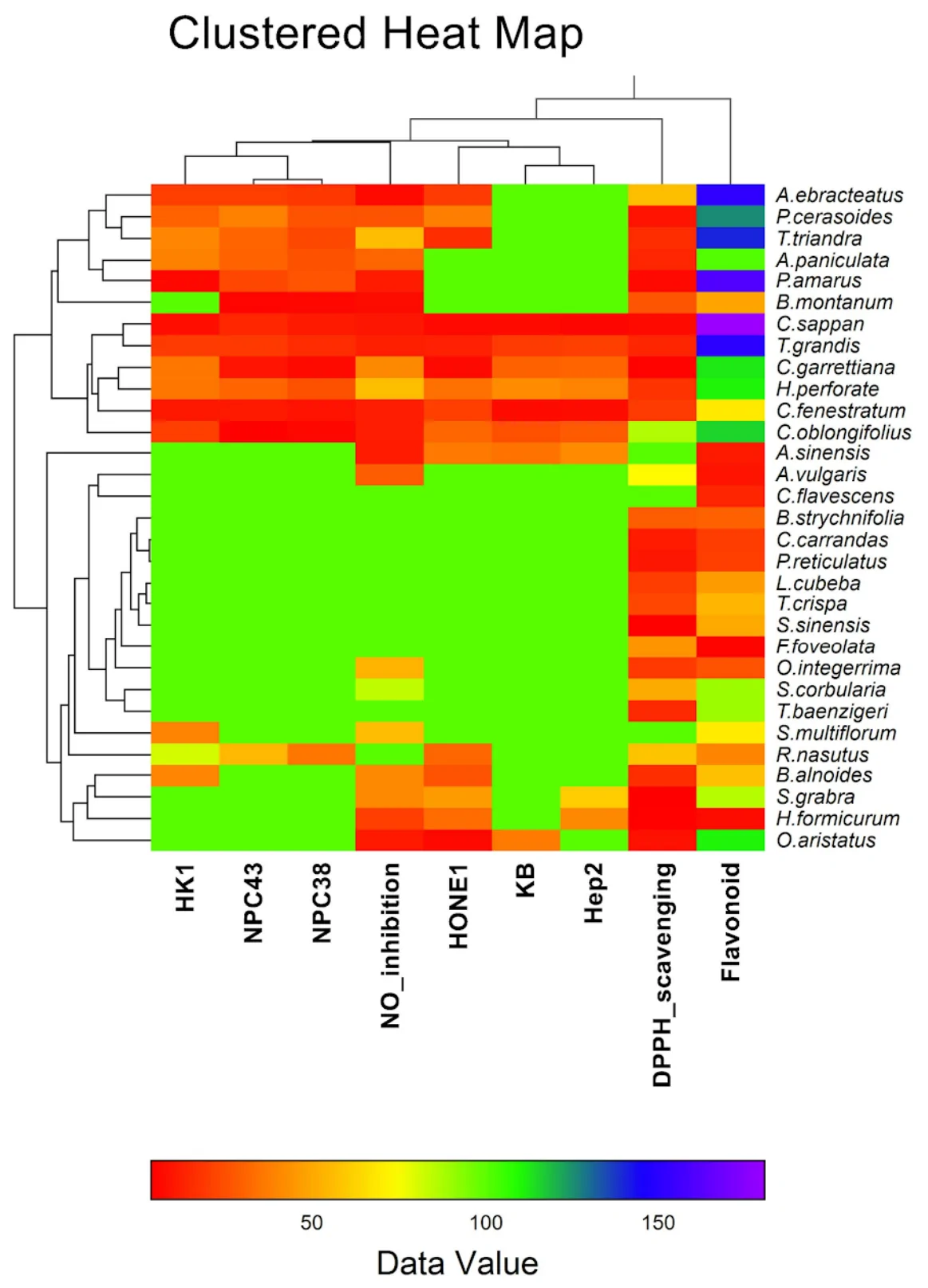
Clustered heatmap of cytotoxic and biological activity profiles of Thai ethnobotanical plant extracts from Kampramong’s anticancer remedies.

Traditional Thai remedies (TTR) are an excellent source of ethnobotanical plants that are safe and effective against many diseases, including malignancies. Therefore, Kampramong’s anticancer remedies were selected to identify potential plants that could be cytotoxic against all URT malignant cells. The result showed that most of the plant components of Kampramong’s anticancer remedies showed cytotoxic effects (Table 1). Furthermore, it was found that *C. sappan*, *C. garrettiana*, *C. fenestratum*, *C. oblongifolius*, *H. perforate*, and *T. grandis* demonstrated cytotoxic effects against all URT malignant cells. Notably, the ethanolic extracts of *C. sappan*, *C. fenestratum,* and *T. grandis* exhibited potent cytotoxic activity against all URT malignant cells with IC_50_ values lower than or close to that of standard cisplatin. Further validation with a heat map dendrogram demonstrated three groups of cytotoxicity. Six out of thirty-three extracts exhibited active cytotoxic activity (IC_50_ < 20 µg/mL) with variable potency. Four plant extracts showed moderately active cytotoxicity (IC_50_ = 20–100 µg/mL); the remaining plants did not show cytotoxic activity (IC_50_ > 20 µg/mL) (Figure 1). Moreover, to evaluate the cytotoxicity of three extracts in two normal epithelial cell lines, including NP460 and HaCaT cells, were selected for examination of safety, the results of these tests showed non-toxicity to normal cells. Collectively, these results indicate that Thai ethnobotany could be an alternative source to treat URT malignancy and potential herbal medicines to prevent URT field cancerization.

To quantitatively benchmark the selectivity of the most active extract against normal cells, the selectivity index (SI = IC_50_ of non-cancerous cells/IC_50_ of cancer cells) was calculated from the IC_50_ values in Table 1. Using the HaCaT IC_50_ (96.39 µg/mL) as the reference, *C. sappan* gave SI values of 13.4 (HK1), 16.5 (KB), 13.4 (HONE-1), 9.6 (NPC38), 7.1 (NPC43) and 20.5 (HEp2); against the NP460 line, where the IC_50_ exceeded 100 µg/mL, the SI values exceeded 10 times (SI > 10) for every malignant cell line tested. These SI values confirm that *C. Sappan*’s cytotoxicity is not attributable to general cellular toxicity and quantitatively support its selectivity for URT malignant cells over non-cancerous controls.

**Figure 2:**
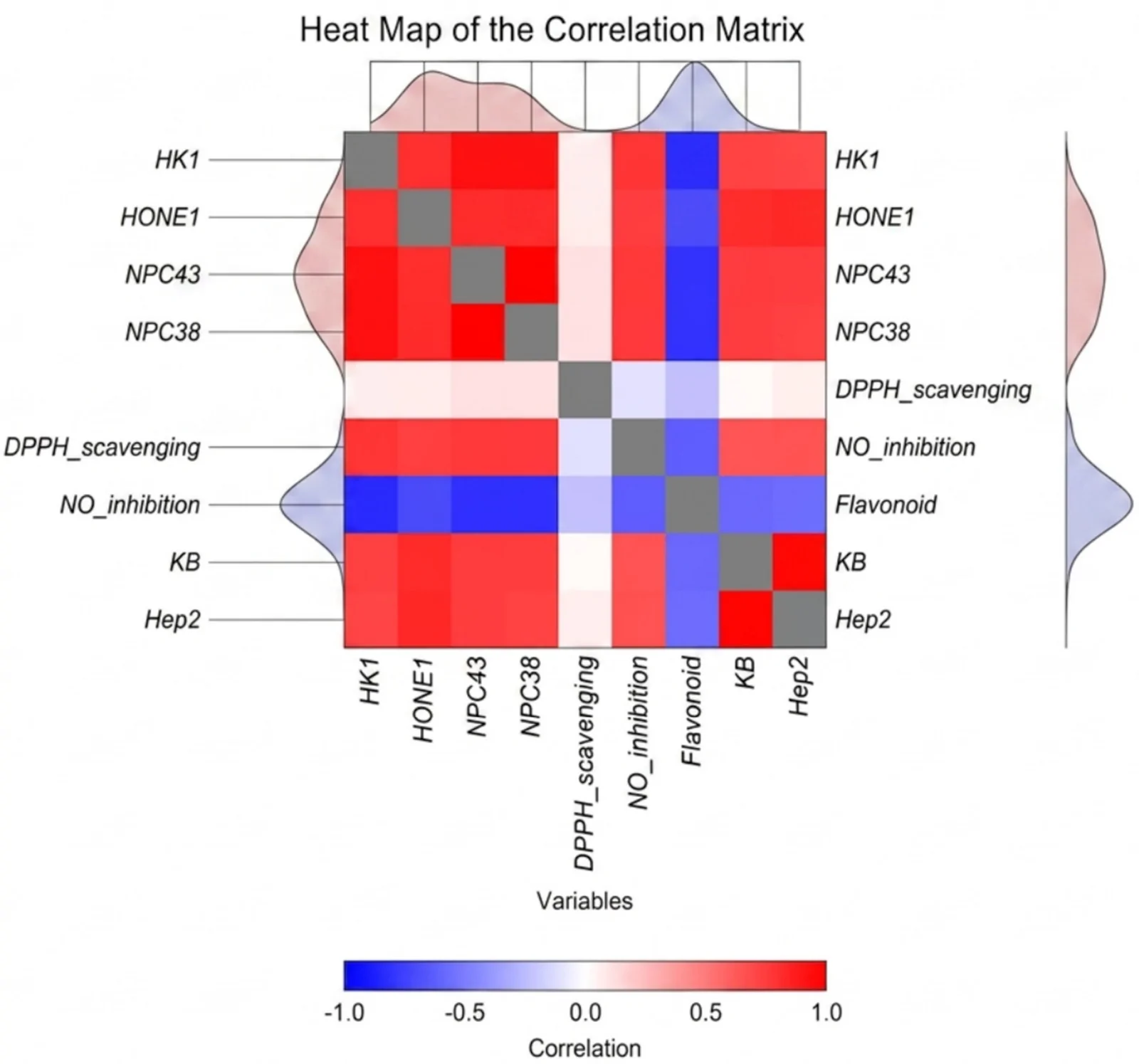
Correlation heatmap of cytotoxic activity, antioxidant activity, anti-inflammatory activity, and total flavonoid content in Kampramong’s anticancer remedy extracts. The color scale represents the Pearson correlation coefficient ranging from −1.0 to 1.0, as shown in the scale bar. Red indicates a strong positive correlation, blue indicates a strong negative correlation, and grey/white indicates no or very weak correlation. Grey blocks along the diagonal indicate self-correlations, which are always equal to 1.0 and were masked to reduce visual distraction and facilitate interpretation of correlations between different variables.

**Figure 3:**
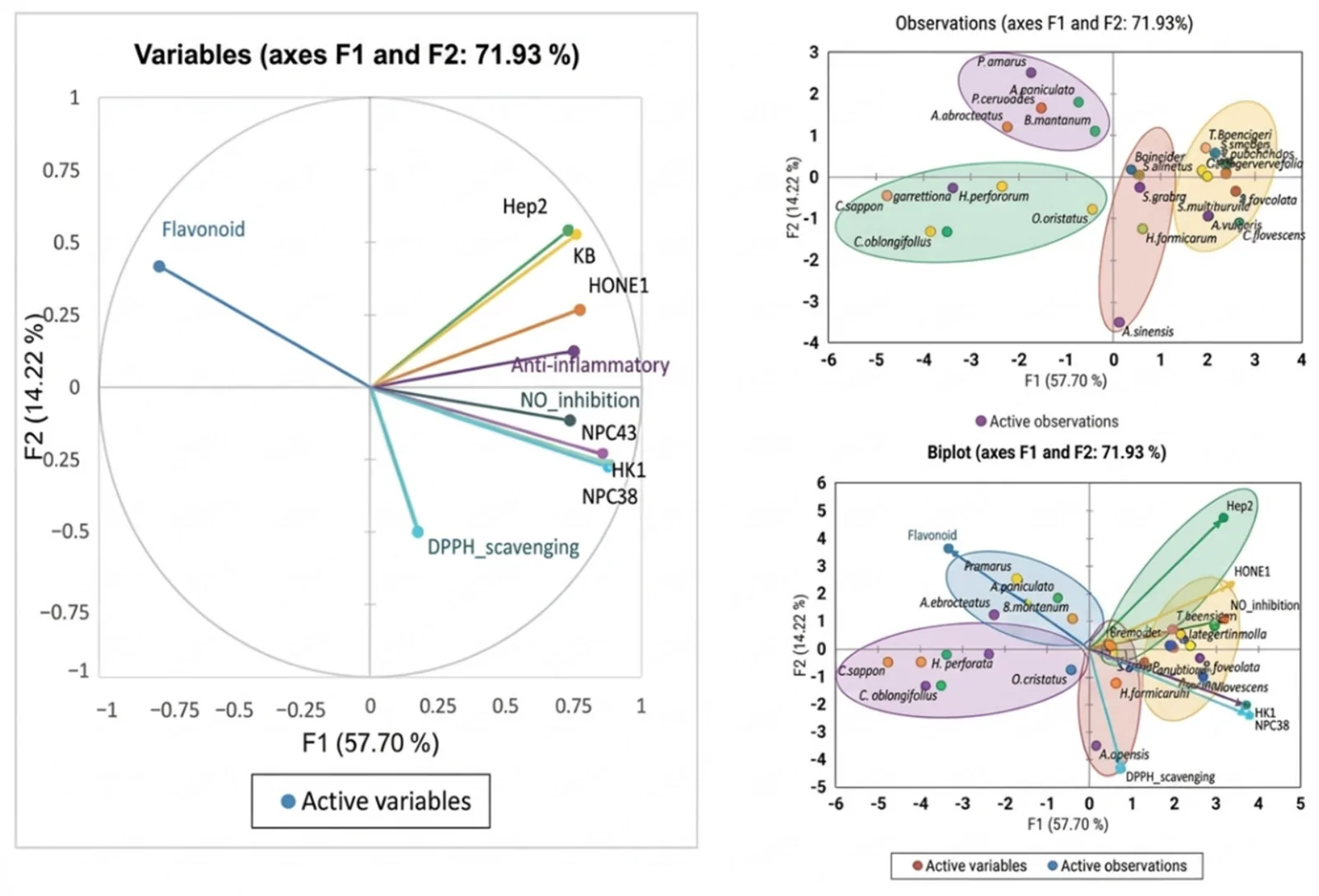
Principal component analysis biplot showing variable loadings and observation scores for cytotoxicity, antioxidant activity, anti-inflammatory activity, and total flavonoid content of Kampramong’s anticancer remedy extracts (F1–F2: 71.93%).

From Figure 1, a summary of Cytotoxic activity was demonstrated by a clustered heat map of Thai plant ingredients of Khampramong remedy and their biological activity. Figure 2. Shows the matrix of heat maps of cytotoxic activity with different types of malignant cells and the biological activities involved in reducing the risk of the upper respiratory tract. Figure 3 is a loading plot of the contribution of the variables’ cytotoxic activity, antioxidant activity, and total flavonoid content in the two-dimensional space formed by the analysis of principal component analysis.

### 2.3. Antioxidant Activity of Plant Extracts Determined by DPPH Radical Scavenging Assay

Table 1 shows the antioxidant activity of plant components in Kampramong’s anticancer remedies. The results found that thirteen plants showed good activity (EC_50_ < 15 µg/mL), nine plants showed medium activity (EC_50_ = 15–35 µg/mL), and nine plants showed low activity (EC_50_ > 35 µg/mL). Lower EC_50_ values indicate greater antioxidant potency.

The findings showed that *S. grabra*, *H. formicarum*, and *S. chinensis* had high antioxidant activity (EC50 values of 3.12 ± 0.11, 3.47 ± 0.23, and 3.70 ± 0.17 µg/mL, respectively), whereas *A. sinensis* and *C. flavescens* had low antioxidant activity with EC_50_ values of 95.65 ± 0.25 and 98.00 ± 0.10 µg/mL, respectively (Table 1). Next, these results were analyzed with PCA for their correlation with other biological factors, as presented in Figure 2. PCA indicated that DPPH scavenging was only weakly associated with URT cancer-cell cytotoxicity.

### 2.4. Total Flavonoid Content

To investigate the correlation between flavonoid content and cytotoxic effect on URT malignancy, the colorimetric aluminum chloride (AlCl3) assay was performed to determine TFC. TFC varied markedly among extracts (Table 1), with the highest value in *O. grandiflorus* (227.95 ± 5.69 mgQE/g) and the lowest in *A. sinensis* (15.15 ± 0.93 mgQE/g). PCA showed that TFC was the strongest variable associated with cytotoxicity and NO-inhibitory activity., as described in Figure 2. PCA analysis demonstrated that TFC had the strongest positive correlation with anti-inflammatory and cytotoxic activities.

### 2.5. Anti-Inflammatory Activity by LPS-Induced NO Production in RAW 264.7 Cells

It was determined that the plant components in Kampramong’s anticancer remedies vary in NO inhibition activity, determined via the Griess assay following LPS-stimulated nitric oxide production (Section 4.7) (Table 1). The highest anti-inflammatory activity was obtained from *A. ebracteatus*, with an IC_50_ value of 5.90 ± 1.55 µg/mL. Ten other plants showed strong anti-inflammatory activity, including *B. montanum*, *C. sappan*, *O. grandifloras*, *P. amarus*, *A. sinensis*, *C. fenestratum*, *C. oblongifolius*, *T. grandis*, *H. formicarum*, and *P. cerasoides* (IC_50_ values of 6.06 ± 0.49, 9.35 ± 0.78, 10.00 ± 0.15, 10.48 ± 1.34, 10.52 ± 2.31, 10.84 ± 1.28, 10.95 ± 4.78, 11.15 ± 3.58, 20.05 ± 1.65, and 25.49 ± 2.52 µg/mL, respectively).

The cytotoxicity of these extracts against RAW 264.7 macrophages was also tested using an MTT assay. The extracts did not exhibit significant cytotoxicity at the concentrations that inhibited NO production. The correlation between anti-inflammatory and antioxidant activity and TFC was analyzed and presented in Figure 2.

Overall, NO inhibition aligned more closely with TFC and cytotoxicity than DPPH scavenging, supporting a stronger contribution of flavonoid-associated anti-inflammatory activity to the observed bioactivity profile.

### 2.6. Multivariate Analysis; PCA and HCA

The main objective of PCA is to identify substantively significant factors in the biological process of carcinogenesis that involves multiple factors. Multivariate analysis was therefore used to identify factors associated with the observed biological activities. PCA of the auto-scaled nine-variable bioactivity matrix in this study retained two components, explaining 71.92% of the total variance (PC1 = 57.70%, PC2 = 14.22%; Table 2). Component selection followed the dual criterion of eigenvalue > 1 and scree-plot inspection [12,13]. PC1 was driven by total flavonoid content (loading r = −0.94) and by cancer-cell IC_50_ values across all six URT cell lines (loadings r = +0.72 to +0.88). PC2 was dominated by DPPH EC_50_ (loading r = −0.61), indicating a separate antioxidant-related axis that did not align with PC1. This orthogonal loading pattern indicates that, in the present dataset, strong DPPH scavenging and strong cancer-cell inhibition reflect distinct bioactivity dimensions rather than a single antioxidant-dependent mechanism. The communality (h^2^) of TFC was high (0.93), and the communalities of the six cancer-cell IC_50_ variables ranged from 0.64 to 0.89, supporting the appropriateness of the retained two-component solution.

HCA (Euclidean distance, Ward’s linkage; 1000-replicate bootstrap stability assessment [14,15]) grouped *C. sappan*, *C. fenestratum*, and *T. grandis* into a single cluster with high bootstrap support; these three extracts shared the highest TFC values (68.95–227.95 mg QE/g), lowest IC_50_ values (4.71–13.60 µg/mL), and SI ≥ 10 against both NP460 and HaCaT. This reproducible HCA pattern was consistent with the PCA findings, and the convergence of the two unsupervised methods supports the interpretation that the leading extracts share a high-TFC, low-IC_50_ profile associated with NO inhibition rather than with DPPH scavenging alone.

The same multivariate structure informed the prioritization of *C. sappan* for the downstream docking workflow. *C. sappan* consistently ranked as the most promising phenolic-rich ingredient across the full screening framework (cytotoxicity against six URT cancer cell lines, SI ≥ 10 against NP460 and HaCaT, clustering with high-TFC and low-IC_50_ extracts in PCA/HCA, and reproducible NO-inhibitory activity) and was therefore carried forward to the STRING-based PPI analysis described next. Figure 3 illustrates the PC. The complete variable loadings for PC1 and PC2 are provided in Table 2.

A graphical representation of the different groups was created using HCA to measure the proximity between the samples. A dendrogram is shown in Figure 1 as a result of applying HCA to the PC matrix. The samples clustered into two major groups. PCA and HCA confirm a correlation between the cytotoxic activity of ethnobotanical plants used in these studies and URT cancer cells. TFC and anti-inflammatory activity, as reflected by NO inhibition, were strongly associated with cytotoxic activity against URT cancer cells, whereas antioxidant activity showed a weaker association.

Furthermore, PCA suggests that the extracts with higher flavonoid contents tended to exhibit stronger NO inhibitory and cytotoxic effect. These findings indicate that flavonoid compounds in these plants are key contributors that should be investigated by a detailed molecular docking analysis.

### 2.7. Molecular Docking Study

**Figure 4:**
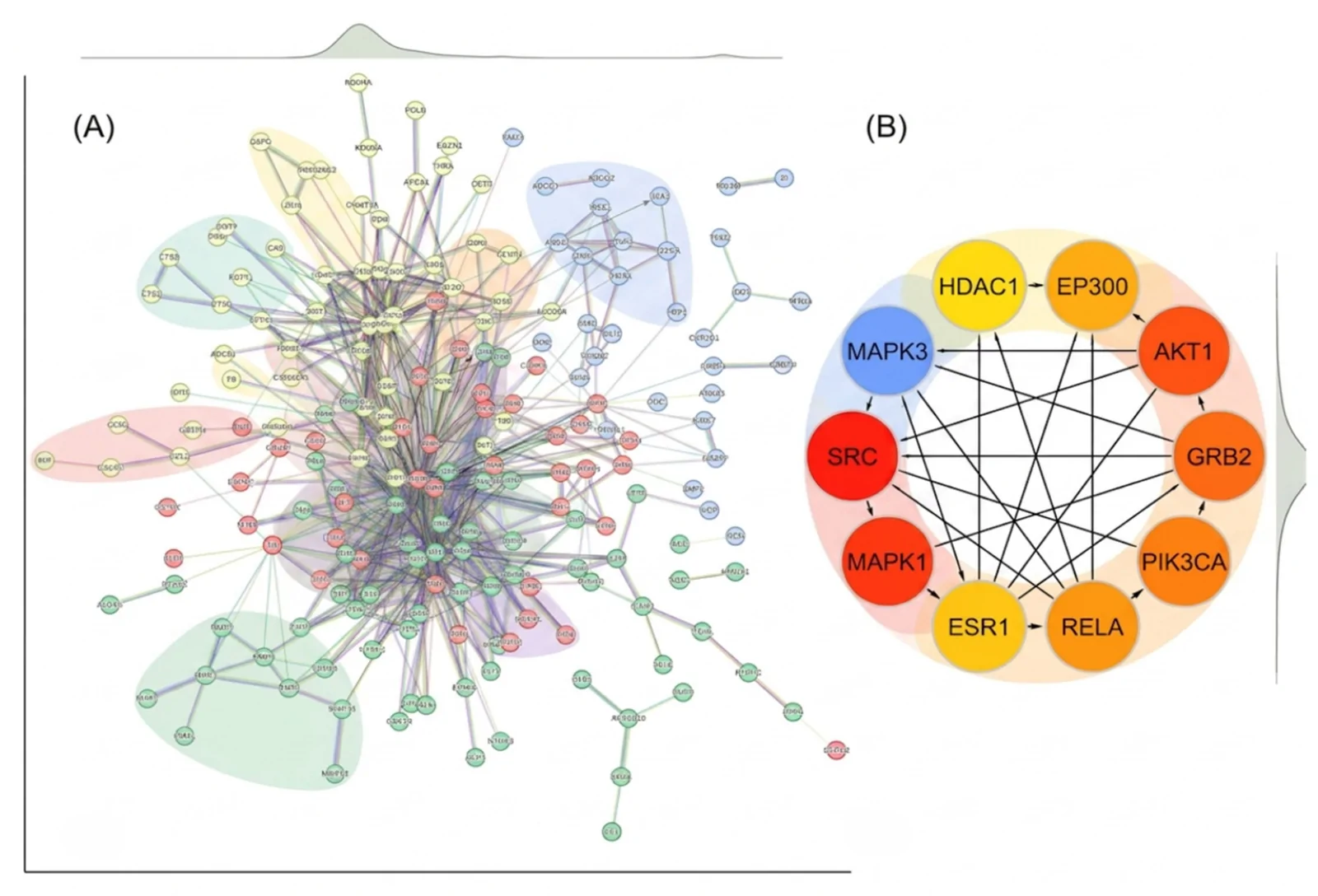
Protein–protein interaction network of URT malignancy-associated targets used for docking prioritization. (**A**) STRING-derived network showing functional relationships among cancer-related proteins. More highly connected nodes represent proteins with greater network centrality in the analyzed target set. (**B**) Top-ranked hub proteins were selected for exploratory docking owing to their central involvement in proliferation-, survival-, and MAPK-related signaling pathways. Darker red indicates a high-er-ranked hub protein with greater network importance.

In the present analysis, The NPC-related protein network is presented in Figure 4A the top 10 enriched proteins are presented in Figure 4B The top 5 proteins, MAPK3, MAPK1, SRC, AKT1, and GRB2, in the STRING network ranked by the degree method are presented in Table 3, and were selected for additional molecular docking studies. *C. sappan* was selected for exploratory docking based on the integrated experimental framework rather than on docking alone. Across the full screening framework-cytotoxicity against six URT cancer cell lines, SI ≥ 10 against NP460 and HaCaT, clustering with high-TFC and low-IC_50_ extracts in PCA/HCA, and reproducible NO-inhibitory activity-*C. sappan* consistently ranked as the most promising phenolic-rich ingredient. STRING-based PPI analysis then prioritized five network-relevant targets for exploratory assessment: MAPK3 (node score 46), SRC, MAPK1, AKT1, and GRB2. These five proteins converge on MAPK activation (MAPK1/3), SRC-associated proliferation, AKT1-driven survival, and GRB2-mediated EGFR–RAS–MAPK relay, defining the docking-target panel used in the following subsections.

**Figure 5:**
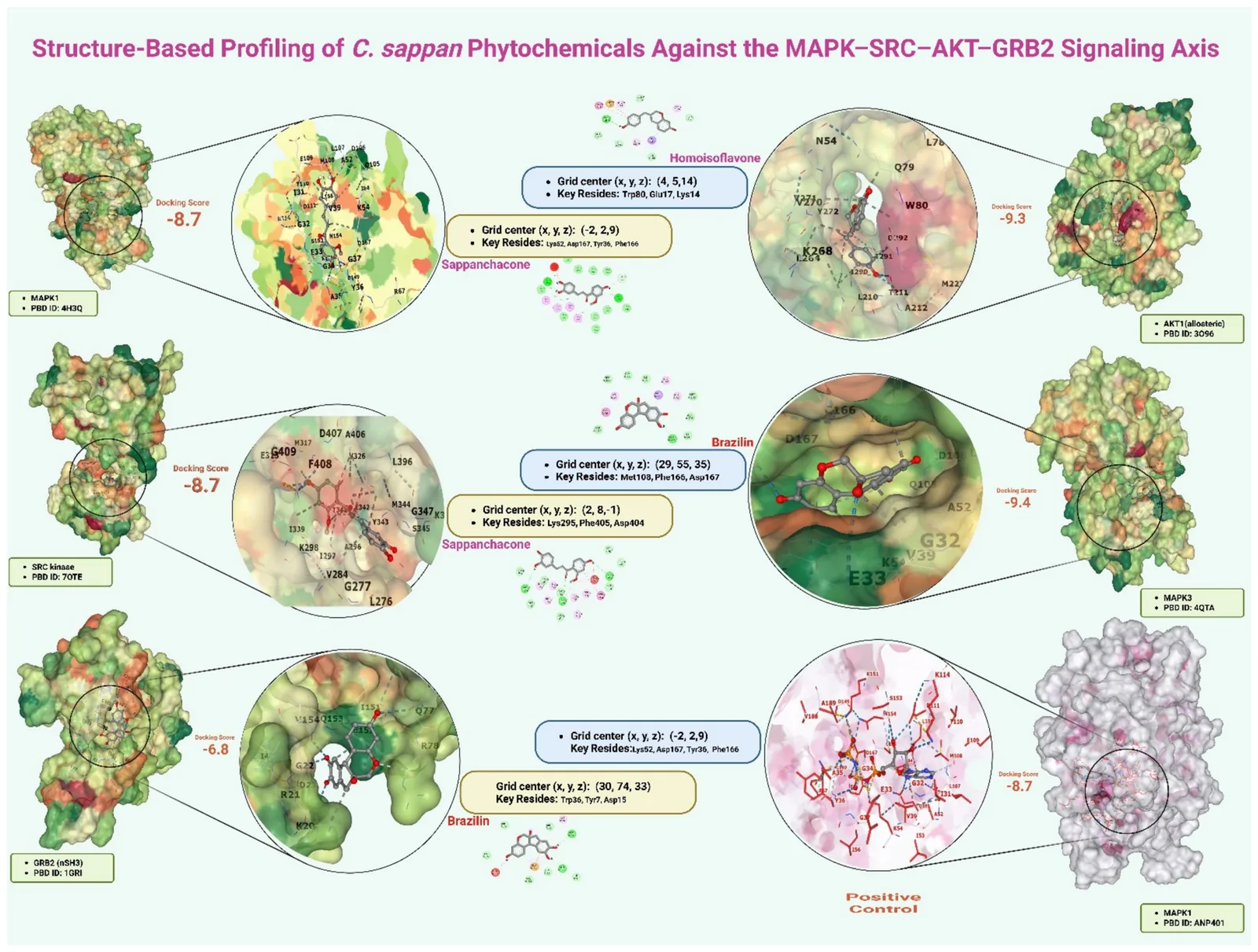
Predicted docking scores of brazilin and sappanchalcone against MAPK1, MAPK3, SRC, AKT1, and GRB2. More negative values indicate more favorable predicted poses within the Vina scoring function. These scores are useful for ranking computational hypotheses but should not be interpreted as experimentally measured binding free energies or confirmed target inhibition.

The drug-likeness approach (Lipinski’s rule of five) was used to screen bioactive compounds of *C. sappan*, which have ADME (Absorption, Distribution, Metabolism and Excretion) restrictions while obtaining an oral bioavailability score >0.50. As presented in Table 4, an online program, SwissADME, was used to explore the drug-likeness properties of identified compounds. Brazilin and sappanchalcone were selected as representative *Caesalpinia sappan* constituents for the molecular docking study because they link the phytochemical profile of the active extract with the multivariate findings. Brazilin was included as a major reported marker compound of *C. sappan*, whereas sappanchalcone represented the flavonoid-associated activity, highlighted by TFC, NO inhibition, and PCA/HCA. Both compounds also satisfied key SwissADME criteria, including Lipinski compliance and predicted oral bioavailability > 0.50. Their preliminary docking profiles and binding sites on the target protein against MAPK1, MAPK3, SRC, AKT1, and GRB2 were analyzed. Binding energies below −8.0 kcal/mol were considered indicative of strong binding affinity. The molecular docking profile is presented in Table 5 and Figure 5.

A summary of the molecular docking results for key phytochemicals derived from *C. sappan* against the major target proteins in the MAPK1–MAPK3–SRC–AKT1–GRB2 panel is presented in Table 6.

Molecular docking showed that brazilin exhibited stronger binding affinity than sappanchalcone for four of the five target proteins, particularly GRB2 (−9.6 kcal/mol), SRC (−9.0 kcal/mol), MAPK1 (−8.6 kcal/mol), and AKT1 (−8.1 kcal/mol), all of which met the predefined strong-binding threshold of ≤−8.0 kcal/mol. In contrast, sappanchalcone showed slightly stronger binding to MAPK3 than brazilin (−7.8 vs. −7.6 kcal/mol), although neither value reached the strong-binding threshold. These findings suggest that brazilin may be the principal multi-target compound, while sappanchalcone may provide complementary activity, supporting a potential multi-compound, multi-target mechanism of *C. sappan*.

#### 2.7.1. Subgroup Analysis of Selected Plant Extracts from In Vivo Studies: Cross-Activity Profiling of Bioactivity Outcomes

Following the target prediction analysis described in Section 2.7.1, molecular docking was performed to evaluate the binding potential of the selected bioactive compounds against the core protein targets; the corresponding binding energies, interacting residues, Table 7. NO inhibition, DPPH antioxidant activity, and total flavonoid content of selected extracts.and interaction types are presented in Table 7 and Table 8.

The data from the above table demonstrated that the stronger activity of whole *C. sappan* extract than isolated brazilin is consistent with a possible multi-compound contribution, but additivity or synergy cannot be concluded from these data alone, not as evidence of direct binding or pathway modulation [17,18,19].

Taken together, the integrated results allow a cautious hypothesis that *C. sappan* may influence a multi-target signaling context involving MAPK1, MAPK3, SRC, AKT1, and GRB2, but this hypothesis remains to be verified experimentally [20,21,22,23].

#### 2.7.2. Receptor Validation and Docking Protocol

Each receptor was validated by redocking its co-crystallized ligand under the same SeamDock and CB-Dock2 settings used for phytochemical docking. Docking was accepted when the heavy-atom RMSD between the redocked and experimental poses was ≤2.0 Å [16,24,25,26].

All five targets (MAPK1/4H3Q, MAPK3/4QTA, SRC/2H8H, AKT1 allosteric/3O96, GRB2/1GRI) passed this threshold. Grid boxes were centered on the co-crystallized ligand or annotated active-site residues with 15 Å padding, and exhaustiveness was set to 32 to balance run-time against docking-score reproducibility [17,27]. Ligands were prepared in Open Babel v3.1.1 using the Universal Force Field with 200 steepest-descent geometry-optimization steps [17,28]. Throughout this section, docking scores are reported as comparative Vina-based predictions in kcal/mol and are not interpreted as experimental binding free energies [18,19].

#### 2.7.3. Docking Performance Across the Target Panel

Brazilin and sappanchalcone were selected as representative *C. sappan* constituents for exploratory docking because they connect the phytochemical profile of the lead extract with the multivariate findings. Brazilin was included as a major reported marker compound of *C. sappan* [29], whereas sappanchalcone was used to represent the flavonoid-associated activity highlighted by TFC, NO inhibition, and PCA/HCA [30,31,32]. Both compounds satisfied key SwissADME criteria, including Lipinski compliance and a predicted bioavailability score of 0.55.

Brazilin generated favorable cross-platform predictions at MAPK1 (−8.7/−8.5 kcal/mol), AKT1 (−8.1 kcal/mol), and MAPK3 (−9.4/−9.1 kcal/mol), whereas sappanchalcone generated a favorable cross-platform prediction at SRC (−6.9 to −8.5 kcal/mol, depending on platform). MAPK3 scores showed the cross-platform variability typically observed for kinase ATP-site targets [16,18,19], and the GRB2 ΔG of −9.6 kcal/mol for brazilin was the strongest signal in the panel. These observations were used only to propose ligand–target interaction hypotheses for future validation and should not be interpreted as evidence of binding affinity, pathway inhibition, or per-target selectivity [17,19]. The detailed docking summary, including estimated Ki values, RMSD controls, and CB-Dock2 vs. SeamDock cross-platform consistency, is provided in Table 5.

#### 2.7.4. ADME and Drug-Likeness Profile

The drug-likeness properties of brazilin and sappanchalcone are summarized in Table 4. Both compounds satisfied Lipinski’s Rule of Five and Veber’s criteria, showed predicted gastrointestinal absorption, and presented a predicted bioavailability score of 0.55. These ADME properties support oral exposure of the major *C. sappan* phytochemicals in further experimental evaluation; however, the SwissADME-predicted profile of polyphenolic constituents should be interpreted as a screening estimate rather than as confirmed pharmacokinetic behavior [17,18,19].

#### 2.7.5. Cytotoxicity, Selectivity, and Bioactivity Cross-Walk

The cross-walk between experimental cytotoxicity and the docking-derived ΔG anchors is summarized in Table 6 and Table 7. *C. sappan* showed higher cytotoxic activity than cisplatin against HK1, KB, HONE-1, NPC38, and HEp2, and comparable activity against NPC43. The whole *C. sappan* extract was also more selective than isolated brazilin in this panel, in agreement with a possible multi-constituent contribution [4]. However, additivity versus synergy cannot be concluded from these data alone and should be evaluated in bioassay-guided fractionation and Chou–Talalay combination-index analysis [33].

*C. sappan* also exhibited strong DPPH activity (EC_50_ = 5.75 ± 0.86 µg/mL), NO inhibition (IC_50_ = 9.35 ± 0.78 µg/mL), and high total flavonoid content (180.33 ± 0.95 mg QE/g), supporting a phenolic-rich antioxidant and anti-inflammatory profile [5,34,35,36]. PCA showed that NO inhibition and DPPH scavenging loaded onto partially distinct axes, consistent with the interpretation that the anticancer-relevant activity of *C. sappan* is unlikely to depend on antioxidant capacity alone [12,13]. The Table 7 cross-walk should therefore be interpreted as an exploratory structural hypothesis and as a guide for deeper validation, not as evidence of direct binding or pathway modulation [17,19].

### 2.8. Analysis of Core Target Protein on Median Survival of HNC Patients

**Figure 6:**
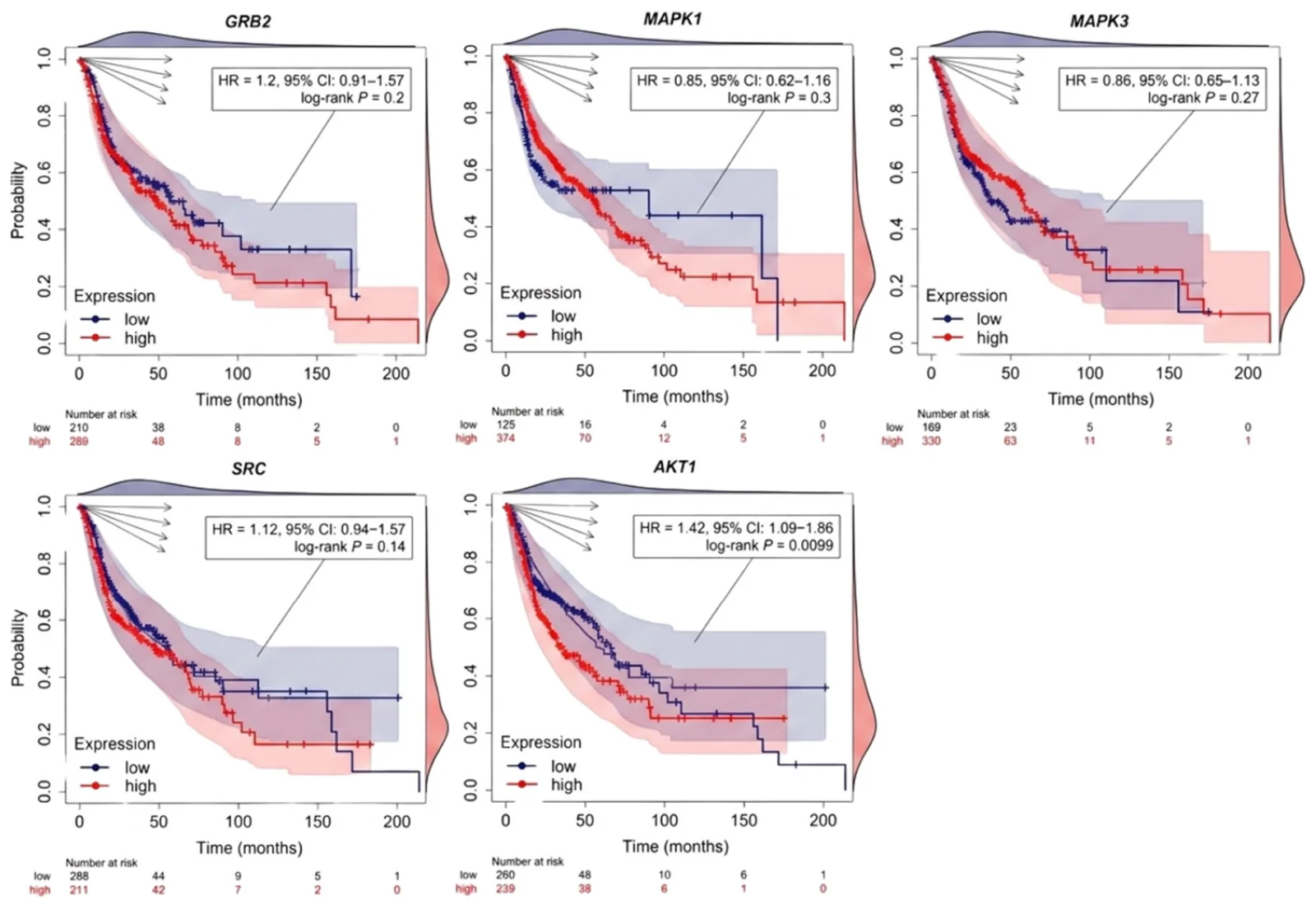
Kaplan–Meier overall survival analysis of head and neck cancer patients stratified by MAPK1, MAPK3, SRC, AKT1, and GRB2 expression.

Figure 6 displays the Kaplan-Meier overall survival curves for HNC patients based on the expression levels of the five core target genes (GRB2, MAPK1, MAPK3, SRC, and AKT1). The full Kaplan-Meier statistics, sourced from integrated TCGA and GEO head and neck squamous cell carcinoma cohorts (*n* = 522 screened; *n* = 499 with complete survival and expression data for all five genes; median-expression cut-off for high/low stratification). Briefly, high AKT1 expression was significantly associated with poorer overall survival (HR = 1.42, 95% CI: 1.09–1.86, log-rank P = 0.0098, *n* = 260 low- vs. 239 high-expression patients), whereas GRB2 (HR = 1.20, 95% CI: 0.91–1.57, P = 0.2000, *n* = 210/289), MAPK1 (HR = 0.85, 95% CI: 0.62–1.16, P = 0.3000, *n* = 125/374), MAPK3 (HR = 0.86, 95% CI: 0.65–1.13, P = 0.2700, *n* = 169/330), and SRC (HR = 1.22, 95% CI: 0.94–1.60, P = 0.1400, *n* = 288/211) did not reach statistical significance. These results indicate that, among the five core target genes, AKT1 expression alone carries independent prognostic value for overall survival in this cohort, while the remaining four genes should be interpreted as mechanistically relevant docking targets rather than independent prognostic biomarkers.

## 3. Discussion

HNC is one of the most common cancers in Thailand and is a common cause of treatment failure, mainly due to field cancerization [21,37]. This is the first study to use a group of respiratory cancer cells as a field cancerization model for the URT cancer study. The present investigation examined the 95% ethanolic extract from the plant components of Kampramong’s anticancer remedies because 95% ethanolic extraction can provide a wider range of polarities of the ingredients [38]. Here, the cytotoxic screening showed that three plant extracts (*C. sappan*, *C. fenestratum*, and *T. grandis*) exhibited potent cytotoxic effects against all URT malignant cells, while also being safe to non-cancerous cells. Therefore, it is reasonable that these plant extracts could be a potential source for preventing and treating URT cancers. The other plant extracts show moderate to no activity, despite being the composition of the anticancer formulations; however, these plants may be active in other cancers or have other supportive or synergistic effects for cancer treatment, such as antioxidant and anti-inflammatory activities or reducing the toxicity of other plant components in the remedies.

The biological activity results involved in HNC prevention showed that all promising plants examined meet this activity. PCA reveals that TFC is the highest correlated factor with anti-inflammatory and cytotoxic activity, but antioxidant is not. Antioxidant compounds generate cytotoxic activity in several ways by improving ROS scavenging enzymes, targeting NADPH oxidase, and manipulating nitric oxide compounds [14]. In contrast, this result showed that most extracts had high antioxidant activity but low cytotoxic activity in URT cancer cells, possibly due to ethanolic extracts that contained highly antioxidant compounds other than cytotoxic compounds. Osman et al. [36]. reported that cytotoxic mechanisms occur in different ways depending on the radical scavenging activity and the structure of these other compounds.

The lower correlation between cytotoxic and antioxidant activity may be due to the fact that cytotoxic activity is derived not only from antioxidant levels but also from other inhibitory effects through various signaling pathways. Another reason is that phenolic compounds are known to have contradictory behavior characterized by antitumor and protumor activities, depending on their chemical structure. The structure specificity of the phytochemical compounds, which have an anticancer effect and are necessary for damage to cancer cells by oxidative stress, could be the other reason for the weak correlation finding [36]. However, DPPH radicals scavenging assays are more suitable for lipophilic antioxidants, other antioxidant assays, such as ABTS, should be further investigated. DPPH is a stable free radical that is reduced predominantly through single electron transfer (SET) and, to a lesser extent, hydrogen atom transfer (HAT), and it reacts more readily with lipophilic, hydrogen-donating phenolics such as those enriched in these extracts. ABTS, by contrast, is soluble in both aqueous and organic media and captures a broader range of hydrophilic and lipophilic antioxidants, so the two assays are not expected to rank extracts identically; comparing them in future work would help determine whether the weak DPPH-cytotoxicity correlation reported here reflects a genuine mechanistic dissociation or an assay-selection effect. Moreover, chemical analyses do not necessarily reflect the cellular effects of antioxidant compounds. Consequently, cell-based tests for cellular antioxidants could be further investigated [12].

TFC of the extracts was highly related to the cytotoxic activity. As in other studies, the data show that the intake of flavonoids is associated with a lower risk of URT cancer. These associations suggest a protective effect of flavonoids against URT malignancy, making it a potential risk reduction strategy [13].

In the present analysis, *C. sappan* is considered the most active cytotoxic agent, with IC_50_ values ranging from 4.0–10.0 µg/mL against all URT carcinoma cells. Moreover, *C. sappan* extract was found to have a stronger cytotoxic effect than the pure brazilin compound. This is likely due to the synergistic effect of the compounds in its crude extract, which has been reported in other studies. Because the crude extract is a mixture rather than an isolated compound, its overall target selectivity reflects the combined, and potentially competing, binding behaviour of brazilin, sappanchalcone, and other co-occurring constituents rather than the selectivity profile of brazilin alone; synergistic interactions among these compounds may broaden target engagement and enhance potency, whereas antagonistic interactions could in principle reduce affinity for a given target relative to the pure compound. This distinction is relevant to interpreting the docking results below, which were generated for the two isolated marker compounds and therefore represent an idealised, single-ligand approximation of the more complex mixture actually tested in the cytotoxicity assays [34]. Furthermore, cytotoxic activity is 2–3 times higher than cisplatin. These results suggest that *C. sappan* can be used as a chemotherapeutic agent to prevent and treat URT malignancy without harming normal cells. Therefore, *C. sappan* was selected for the underlying mechanism study as it has the highest cytotoxic activity and presents all the properties to reduce the risk of URT malignancy.

The five proteins, MAPK3, MAPK1, SRC, AKT1, and GRB2, were selected through the PPI network for the molecular docking study. Brazilin was selected as the main bioactive compound found in *C. sappan* and present in the highest amount [39]. Sappanchalcone, in addition, was selected based on the PCA result, which suggests that flavonoids were the main factor involved in anti-URT cancers, and sappanchalcone is the main bioactive flavonoid found in *C. Sappan* [40]. Therefore, these compounds were selected for a further molecular docking study to visualize the virtue binding site, which could bind to the active protein. The result is shown in Figure 5. Brazilin and sappanchalcone showed a high affinity with a low binding energy of less than −8.0. indicating that *C. sappan*, rich in brazilin and sappanchalcone, could be used to treat URT malignancy. Not only specific for one protein but also targeted for other proteins involved in URT carcinogenesis. Molecular docking demonstrates the benefit of the compound found in *C. sappan* as a potential source for URT treatment and prevention. Interestingly, the ADME parameters indicate the potential role of these two compounds as a source for drug development, as they meet all the parameters of drug-like properties. In general, these results add to the growing body of evidence that these five proteins are a potential target of *C. sappan* for the treatment of URT cancer.

The favourable binding affinities to be 0.55 bioavailability score in Table 4 observed as consistent with the chemical structures of the two marker compounds: brazilin is a homoisoflavonoid bearing a catechol-type dihydroxyphenyl ring together with additional hydroxyl substituents, while sappanchalcone is a hydroxylated chal-cone; in both cases, the multiple hydroxyl groups are well positioned to form hydrogen bonds with polar residues in the target binding pockets, while the fused hydrophobic ring systems fa-vour van der Waals contacts with adjacent nonpolar residues. This combination of hydrogen-bonding and hydrophobic interactions offers a structural rationale for why brazilin and sap-panchalcone, rather than less-hydroxylated or purely aliphatic constituents of the extract, emerged as the dominant contributors to the predicted multi-target binding profile, and is consistent with their corroborating experimental cytotoxic, antioxidant, and anti-inflammatory activity.

The MAPK signal pathway is closely related to tumor biology and regulates normal cell proliferation, differentiation, and apoptosis [41]. MAPK1 is an important gene that promotes cell proliferation, migration, and invasion in cancer [42]. After gene decomposition, it inhibits the proliferation, invasion, and migration of cancerous cells and causes cell apoptosis [43]. The SRC family of non-receptor protein tyrosine kinases plays a crucial role in cancer growth and metastasis [44]. SRC mutations rarely occur in human tumors, so this should be noted [45]. Consequently, increased activity may be caused by abnormal regulation. The AKT/mTOR pathway is a classical signal pathway that maintains energy homeostasis and promotes tumor growth and metastatic development [22]. These signaling pathways are also relevant to treatment resistance in HNSCC. Cisplatin resistance has been associated with NRF2-driven antioxidant responses, downregulation of copper transporters, and reactivation of MAPK/AKT signaling, which intersect with the antioxidant and multi-target activities observed in this study [46]. The predicted interactions of *C. sappan* constituents with MAPK1, MAPK3, SRC, AKT1, and GRB2 therefore provide a rationale for investigating the extract or its active compounds as potential combination partners [47,48,49]. However, molecular docking alone cannot demonstrate reversal of drug resistance or synergistic activity; these possibilities should be confirmed using resistant cancer-cell models and formal combination assays, or require future Western blot or in vitro kinase assays to validate.

*C. sappan*-derived constituents generated predicted docking poses involving MAPK1, MAPK3, SRC, AKT1, and GRB2, proteins related to MAPK activation, SRC-mediated proliferation, AKT1 survival signaling, and GRB2-linked EGFR–RAS–MAPK signaling. These observations are best interpreted as preliminary ligand–target hypotheses and as a guide for deeper validation, because docking scores are comparative structural predictions rather than experimental binding affinities.

Survival analysis provided additional clinical context for this target panel. Among the five docking-prioritized proteins, AKT1 remained significant after Benjamini–Hochberg correction, whereas MAPK1, MAPK3, SRC, and GRB2 showed unadjusted trends. This pattern is biologically plausible because AKT1 functions as a central survival hub that integrates receptor-derived, SRC-related, and MAPK-linked inputs with mTOR-dependent growth and apoptosis-suppression pathways. Thus, AKT1 may represent a clinically relevant convergence point within the broader MAPK–SRC–AKT–GRB2 hypothesis, while the other targets should be treated as exploratory associations requiring validation in local HNC cohorts.

Taken together, the AKT1 survival signal is consistent with a hub-centered network interpretation rather than a single-gene causal conclusion. In the present study, AKT1 should therefore be viewed as a clinically relevant prioritization signal within the broader MAPK–SRC–AKT–GRB2 hypothesis, pending orthogonal functional validation [34,50].

The experimental screening results guided the selection of compounds for exploratory docking. *C. sappan* was prioritized because it combined broad cytotoxicity, high cancer selectivity, high flavonoid content, and NO-inhibitory activity. Brazilin was then selected as a major reported constituent of *C. sappan*, while sappanchalcone was selected to represent the flavonoid-associated activity indicated by TFC and PCA/HCA [34,39]. Thus, molecular docking was used as a follow-up exploratory step derived from the bioactivity and multivariate results, not as an independent or confirmatory mechanism.

The selected target panel is biologically relevant to URT malignancy because MAPK1/3, SRC, AKT1, and GRB2 participate in interconnected growth-factor, proliferation, survival, and MAPK-related signaling pathways. MAPK signaling regulates cell proliferation, differentiation, and apoptosis, SRC contributes to oncogenic signaling and metastatic behavior, and AKT1 links upstream receptor signals to mTOR-dependent growth and survival pathways. These pathway relationships provide biological context for target prioritization but do not establish direct inhibition by *C. sappan* constituents [34,50].

Accordingly, *C. sappan* extract and its representative constituents, brazilin and sappanchalcone, should be interpreted as candidates for further mechanistic investigation rather than confirmed inhibitors of SRC, AKT1, GRB2, or MAPK proteins. The stronger activity of the whole extract compared with isolated brazilin is consistent with a possible multi-compound contribution; however, synergy, additivity, and specific protein inhibition remain unproven. Future work should therefore combine chemical standardization, fractionation or combination studies, kinase or phospho-protein assays, and rescue experiments to evaluate whether the proposed MAPK–SRC–AKT–GRB2-related hypothesis is biologically meaningful.

Experimental validation is essential because docking scores, predicted ΔG values, and estimated *K*_i_ values are structural hypotheses, not confirmed binding or mechanistic evidence [18,19,20,51,52,53,54,55]. Validation plan should include: (i) LC-MS/MS standardization of the active *C. sappan* extract; (ii) fractionation and Chou–Talalay combination-index analysis to test additive or synergistic effects of brazilin, sappanchalcone, and related phenolics; and (iii) kinase activity, phospho-protein profiling, and rescue assays to evaluate the MAPK–SRC–AKT–GRB2-related hypothesis. Each validation assay should include appropriate positive, negative, and vehicle controls; for example, known pathway inhibitors can serve as positive controls, vehicle-treated cells as negative controls, and untreated or non-target pathway conditions as baseline controls. These controls are needed to distinguish true pathway modulation from non-specific cytotoxicity, solvent effects, or assay interference. This framework separates exploratory docking-based prioritization from mechanistic confirmation [21,34].

Taken together, from a mechanistic standpoint, the present integration of cytotoxicity, multivariate clustering, and exploratory docking supports a cautious multi-target hypothesis rather than a single-receptor mechanism [18,19,20].

The cytotoxic potency of the crude *C. sappan* extract (IC_50_, 4.71–13.60 µg/mL) was comparable to or greater than reported values for curcumin (approximately 15–30 µg/mL), but substantially lower than that of paclitaxel (approximately 0.001–0.01 µg/mL). This difference is expected for an unfractionated natural-product extract and highlights the need for bioactivity-guided fractionation for isolated cytotoxic compounds.

## 4. Materials and Methods

### 4.1. Chemicals and Reagents

Fetal bovine serum (FBS) and trypsin-EDTA were purchased from Gibco^®^ (Thermo Fisher Scientific, Waltham, MA, USA). Minimum essential medium (MEM), Dulbecco’s modified Eagle medium (DMEM), Roswell Park Memorial Institute medium 1640 (RPMI-1640), penicillin-streptomycin (P/S), and phosphate buffer saline (PBS) were purchased from Biochrom (Berlin, Germany). Sulforhodamine B (SRB) was purchased from Sigma (St. Louis, MO, USA). Analytical grade ethanol and dimethyl sulfoxide (DMSO) were purchased from RCI LabScan (Bangkok, Thailand). Purified water was prepared using the Milli Q^®^ system from Millipore (Bedford, MA, USA).

### 4.2. Plant Materials and Preparation of Sample Extraction

Kampramong’s anticancer remedies consist of thirty-one different plants. The plants used are as follows: *Acanthus ebracteatus* Vahl, *Andrographis paniculata* (Burm.f.) Wall.ex Nees, *Angelica sinensis* (Oliv.) Diels, *Artemisia vulgaris* Linn., *Baliospermum montanum* (Willd.) Muell. Arg, *Bauhinia strychnifolia* Craib., *Betula alnoides* Buch-Ham.ex G.Don, *Caesalpinia sappan* L., *Carissa carandas* Linn., *Cassia garrettiana* (Craib) Irwin & Barneby, *Coptosapelta flavescens* Korth., *Coscinium fenestratum* (Goetgh.) Colebr., *Croton oblongifolius* Roxb., *Ficus foveolata* Wall., *Harrisonia perforata* (Blanco) Merr., *Hydnophytum formicarum* Jack, *Litsea cubeba* (Lour.), *Ochna integerrima* (Lour.) Merr., *Orthosiphon grandiflorus* Bold., *Phyllanthus amarus* Schumach. & Thonn., *Phyllanthus reticulatus* Poir., *Polyalthia cerasoides* (Roxb.) Benth. ex Bedd. ST, *Rhinacanthus nasutus* (L.) Kurz., *Salacia chinensis* L., *Smilax corbularia* Kunth, *Smilax grabra* Roxb., *Suregada multiflora* (A. Juss) Baill., *Tectona grandis* L.f., *Tiliacora triandra* (Colebr.) Diels, *Tinospora baenzigeri* Forman, and *Tinospora crispa* (L.) Miers ex Hook.f. & Thomson. All the plants used in Kampramong’s anticancer remedies were provided by the Center of Excellence in Applied Thai Traditional Medicine Research, Faculty of Medicine, Thammasat University, Thailand, in 2020. Authentication of plant materials was by comparison with specimens deposited at the herbarium of the Southern Center of Thai Medicinal Plants at the Faculty of Pharmaceutical Sciences, Prince of Songkla University, Songkhla, Thailand. The botanical details, biological activity, and traditional use of Thai ethnobotany components in Kampramong’s anticancer remedy are shown in Table S1.

Plant materials were cleaned, sliced into small pieces, oven-dried at 50 °C, and ground into a fine powder. Each powdered plant material (100 g) was macerated with 95% ethanol (500 mL) for seven days, filtered through a Whatman filter paper No. 1. The filtered extract was dried by rotary evaporator. All extracts were kept in the refrigerator at −20 °C until used.

### 4.3. Cell Lines and Cell Culture

#### 4.3.1. Cancerous and Non-Cancerous Cell Lines and Culture Conditions for Cytotoxicity Assays

Oral squamous cell carcinoma (KB; ATCC no. CCL-17) and laryngeal carcinoma (HEp2; ATCC no. CCL-23) were obtained from the Center of Excellence in Applied Thai Traditional Medicine Research, Faculty of Medicine, Thammasat University, Thailand. Undifferentiated nasopharyngeal carcinoma (HONE-1), well-differentiated nasopharyngeal carcinoma (HK1), EBV-negative recurrent nasopharyngeal carcinoma (NPC38), EBV-positive undifferentiated nasopharyngeal carcinoma (NPC43), and immortalized nasopharyngeal epithelial cells (NP460) were purchased from the Center for Research on Nasopharyngeal Carcinoma, Hong Kong. The immortalized human keratinocyte cell line (HaCaT; CLS Cat no. 300493) was purchased from the CLS cell line service.

HK1, KB, HONE-1, NPC38, NPC43, and HEp2 were used as cell models for URT malignancies. HaCaT and NP460 were used as non-cancerous cell models. HK1 and HONE-1 were maintained in DMEM supplemented with 5% FBS and 5% Newborn Calf Serum (NCS). NPC38 and NPC43 were maintained in RPMI-1640 medium supplemented with 10% FBS and 4 μM Y-27632 dihydrochloride. KB and HEp2 were maintained in RPMI-1640 medium supplemented with 10% FBS. All cell lines were supplemented with 100 U/mL penicillin and 100 mg/mL streptomycin. Cells were cultured in a humidified incubator with 95% air and 5% CO_2_ at 37 °C. NP460 cells were cultured in 1:1 defined keratinocyte serum-free medium (DKSFM) and Epilift medium supplemented with EGF growth factor. HaCaT cells were maintained in DMEM.

#### 4.3.2. Macrophage Cell Line and Culture Conditions for Anti-Inflammatory Assays

RAW264.7 macrophage cell line (ATCC no. TIB-71) was obtained from the Center of Excellence in Applied Thai Traditional Medicine Research (CEATMR), Thammasat University, Pathumthani, Thailand. Cells were maintained in RPMI-1640 medium supplemented with 10% FBS, 100 U/mL penicillin, and 100 mg/mL streptomycin. Cells were cultured in a humidified incubator with 95% air and 5% CO_2_ at 37 °C.

### 4.4. Cytotoxicity Activity by SRB Assay

The 95% ethanolic extracts of ethnobotanical components of Kampramong’s anticancer remedies were evaluated for cytotoxic activity using the SRB assay [56]. Briefly, 100 µL of cells were seeded in 96-well plates with different amounts as follows: HK1: 4.0 × 10^3^ cells/well KB: 1.0 × 10^3^ cells/well, HONE-1: 2.0 × 10^3^ cells/well, HEp2: 1.0 × 10^3^ cells/well, NPC38: 1.5 × 10^3^ cells/well, NPC43: 1.5 × 10^3^ cells/well, NPC460: 5 × 10^3^ cells/well, and HaCaT: 8.0 × 10^3^ cells/well. Incubate the plate for 24 h at 37 °C, 5% CO_2,_ and 95% humidity. Then, 100 µL of a sample solution was added to each well. The final sample concentration in the treated cells was 1, 10, 50, and 100 µg/mL, respectively. DMSO was used as a control solvent at a maximum concentration of less than 0.5% *v*/*v*. The cells were treated for 72 h, and then the mixture in the well was removed and washed with 200 µL of fresh medium. The 96-well plate was incubated for the recovery phase for 72 h, and cells were fixed with 40% trichloroacetic acid (TCA) at 4 °C for 60 min. After that, the 96-well plate was washed with water five times. Finally, the fixed cells in the 96-well plate were stained with 0.4% *w*/*v* SRB in 1% *v*/*v* acetic acid for 30 min, followed by removal of unbound dye with 1% *v*/*v* acetic acid and air-drying. The stained cells were resolved with 100 µL of 10 mM Tris-base, and absorbance at 492 nm was measured using a microplate reader. The % inhibition of cell growth was calculated by the equation shown below, and the IC_50_ values were calculated using the Prism program.

Inhibition (%) = [(OD_control_ − OD_sample_)/OD_sample_] × 100

### 4.5. Antioxidant Activity by DPPH Radical Scavenging Assay

The antioxidant activity of these plant extracts was evaluated by the DPPH radical scavenging assay [57]. Briefly, all samples were dissolved in absolute ethanol and further diluted to 4 concentrations (1, 10, 50, and 100 µg/mL). Each concentration was tested in triplicate. A portion of the sample solution (100 μL) was mixed with an equal volume of 6 × 10^−5^ M DPPH (in absolute ethanol) and allowed to stand at room temperature for 30 min in the dark. The absorbance at 520 nm was measured using a microplate reader. Ascorbic acid was used as the standard antioxidant. The scavenging activity of the samples is the ability to reduce the color intensity of DPPH. The percentage of inhibition was calculated using the following equation, and IC_50_ value was calculated from the Prism program.

Inhibition (%) = [(OD_control_ − OD_sample_)/OD_sample_] × 100

### 4.6. Determination of the Total Flavonoid Content

The total flavonoid content (TFC) was determined using the colorimetric aluminum chloride (AlCl_3_) assay [35]. Quercetin was used as the reference standard. Quercetin is dissolved at 1 mg/mL in absolute ethanol. A standard stock solution is diluted to 7 concentrations in serial dilutions of 240, 120, 100, 80, 60, 40, and 20 µg/mL to establish a standard calibration curve. For sample preparation, ethanolic extracts are dissolved in absolute ethanol. In a centrifuge tube, 500 µL of each sample (1 mg/mL) is mixed with 75 µL of NaNO_2_ (5% *w*/*v*) and 150 µL of AlCl_3_ (10% *w*/*v*) and incubated for 5 min, then 500 µL of 1 M NaOH and 275 µL of distilled water. 200 µL is added to a 96-well microplate and incubated for 30 min at room temperature. Finally, the absorbance is measured at 510 nm. Absolute ethanol was used as blank samples, and the same procedure was followed. TFC is expressed as milligrams of quercetin equivalent (mgQE/g dry extract).

### 4.7. Anti-Inflammatory Activity on Inhibition of NO Production

The inhibitory effect on NO production of RAW 264.7 cells was performed to determine anti-inflammatory activity as previously described [58]. Concentrations of this cell suspension are placed in a 96-well microplate to obtain 1 × 10^5^ cells/well. Cells are incubated at 37 °C and 5% CO_2_ for 24 h. The medium is then replaced with 100 µL of fresh medium containing 1 ng/mL of LPS, and 100 µL of the test samples are added to the same well. The plate is then incubated for 24 h. After that, transfer 100 µL of the supernatant from each well to another unsterile 96-well plate and add 100 µL of Griess reagent to each well to determine NO production. Simultaneously, the cells were tested for cytotoxicity using the MTT assay. Prednisolone was used as a positive control. Measure the optical density of the reaction at 570 nm using a microplate reader. Calculate the inhibitory effect on nitric oxide production by comparing the absorbance using the following equation, and the IC_50_ values were calculated using the Prism program.

Inhibition (%) = [(OD_control_ − OD_sample_)/OD_sample_] × 100

### 4.8. Molecular Docking Study

Software and engine: The molecular docking was analyzed in the SeamDock (v2021.2) program (https://bioserv.rpbs.univ-paris-diderot.fr/services/SeamDock/) (accessed on 1 May 2026), CB Dock2 platform (https://cadd.labshare.cn/cb-dock2/index.php) (accessed on 1 May 2026), and AutoDock Vina (version 1.2.0) scoring function with attractive-cavity search-space identification. Target protein PDB files were retrieved from the PDB database (www.rcsb.org) (accessed on 17 April 2026). The 3D structure of the active compound was recovered from the PubChem database (https://pubchem.ncbi.nlm.nih.gov) (accessed on 17 April 2026). The URT malignancy-targeted protein was collected from the STRING database (https://string-db.org/) (accessed on 17 April 2026), and the active compound was collected from the PharmMapper database (http://www.lilab-ecust.cn/pharmmapper/) (accessed on 13 April 2026). The molecular docking analysis was re-performed using the online platform on 5 June 2026. 

The five target protein structures (PDB IDs 4QTB, 2H8H, 4IZ5, 1UNQ, and 1FYR for MAPK3, SRC, MAPK1, AKT1, and GRB2, respectively) were prepared by removal of co-crystallised water molecules and heteroatoms prior to docking, and ligand three-dimensional coordinates were used as retrieved from PubChem without further manual modification. Docking was performed using the SeamDock web server, which integrates AutoDock, AutoDock Vina, Smina, and Qvina behind a common interface and allows the user to define the docking engine, grid spacing, number of modes, energy range, and exhaustiveness for each run. [Author to confirm and insert for reproducibility: (i) which specific docking engine (AutoDock, AutoDock Vina, Smina, or Qvina) was selected within SeamDock; (ii) the grid spacing, mode number, energy range, and exhaustiveness values used; and (iii) whether ligand protonation/energy minimisation was performed prior to docking or the PubChem 3D structure was docked as retrieved].

### 4.9. Analysis of Core Target Genes with Median Survival of Head and Neck Cancer

The Kaplan-Meier graph (online database by http://kmplot.com) (accessed on 1 May 2026) to analyze the correlation between five key target genes (MAPK1, MAPK3, SRC, AKT1, and GRB2) and the prognosis of HNC patients Public patient datasets were integrated from TCGA (The Cancer Genome Atlas) and GEO (Gene Expression Omnibus) head and neck squamous cell carcinoma cohorts (total screened N = 522; N = 499 with complete survival and expression data for all five genes), with patients stratified into high- and low-expression groups using the median expression value of each gene as the cut-off.

### 4.10. Statistical Analysis

The results of the biological activities were expressed as the mean ± standard error of the mean (SEM) of three independent experiments (*n* = 3). IC_50_ values and EC_50_ values were calculated using GraphPad Prism software version 10.2.3. The correlation between total flavonoid content and cytotoxic activities was plotted using Microsoft^®^ Excel. Hierarchical cluster analysis (HCA) for similarity patterns in the biological activities of the extracts was analyzed using Microsoft^®^ Excel and NCSS^®^ 2025 software. Multivariate analysis was carried out with cluster and principal component analysis (PCA) by NCSS^®^ software. Pearson correlation coefficients (r) and associated *p* values were calculated using scipy. stats. pearsonr. The full correlation matrix with r values is in Figure 3, and selected correlations are quantified in Table 2.

#### Limitations of the Study

Several limitations of the present study should be considered when interpreting the findings. First, the cytotoxic, anti-inflammatory, and antioxidant activities were assessed exclusively in vitro; these results have not yet been confirmed in animal models or clinical settings, and in vivo validation is required before any therapeutic claims can be made. Second, the active compounds were evaluated as crude ethanolic extracts and as the two most abundant isolated constituents, brazilin and sappanchalcone; the extracts were not fractionated further, so the individual contribution of minor constituents to the observed synergistic effect remains unresolved. Third, the molecular docking analysis is a computational prediction of binding affinity and does not constitute direct biochemical or structural proof of target engagement; the proposed multi-target mechanism should therefore be regarded as a hypothesis for future confirmatory studies, such as Western blotting, kinase activity assays, or co-crystallization, rather than as an established mechanism of action.

The present framework has clear limitations that should be addressed before any mechanistic claim is made. First, SwissADME ADME filtering is a screening estimate and does not replace experimental pharmacokinetic confirmation [20]. Second, the higher activity of the whole *C. sappan* extract than isolated brazilin does not distinguish additivity from synergy and should be evaluated in bioassay-guided fractionation combined with Chou–Talalay combination-index analysis. Third, exploratory Vina-based docking should be validated by phospho-Western blot, cell-based kinase assays, and rescue experiments targeting MAPK1/3, SRC, AKT1, and GRB2 before any binding or pathway-modulation interpretation is asserted. Fourth, survival analyses in external HNC cohorts with documented HPV/EBV status, tumor subsite, and treatment history are required to confirm the prognostic associations observed here. Finally, LC-MS/MS standardization and orthogonal in vitro/in vivo validation are required before the multi-target mechanistic hypothesis advanced in this work can be translated into a therapeutic claim.

## 5. Conclusions

The present findings strongly demonstrate that the three Thai ethnobotanical plants of Kampramong’s anticancer remedies (*Caesalpinia sappan*, *Coscinium fenestratum*, and *Tectona grandis*) exerted significant cytotoxicity against all URT cell lines. Correlation, PCA, and HCA studies provided a mechanism to investigate the biological activities of ethnobotanical plants. Importantly, this is the first report based on field cancerization theory showing that Thai ethnobotanical plants have cytotoxic activity against all URT cancers, correlated with flavonoid content and inhibition of NO production. These plants may play a crucial role in treating and preventing the development of URT cancer. Molecular docking reveals the potential role of brazilin and sappanchalcone, the major bioactive compounds found in *C. sappan*, in treating and preventing upper respiratory field cancerization by inhibiting MAPK3, MAPK1, SRC, AKT1, and GRB2 proteins. Taken together, the in vitro cytotoxic, antioxidant, and anti-inflammatory activities of *C. sappan* and the computationally predicted binding of its two principal marker compounds to five carcinogenesis-related proteins converge on a single, internally consistent picture: the extract’s biological potency against URT malignant cells is plausibly driven, at least in part, by multi-target engagement of the MAPK, SRC, and AKT signalling nodes by brazilin and sappanchalcone, acting alongside the antioxidant and anti-inflammatory contributions of co-occurring flavonoids. This integrated view links the experimental bioassay findings to a specific, testable molecular hypothesis rather than treating the cytotoxicity and docking results as separate lines of evidence.

Finally, the mechanistic hypothesis from exploratory docking in this could be concluded that, the STRING-based PPI analysis and exploratory Vina-based docking against MAPK1/3, SRC, AKT1, and GRB2, brazilin generated favorable cross-platform predictions at MAPK1 (−8.7/−8.5 kcal/mol), AKT1 (allosteric −8.1 kcal/mol), and MAPK3 (−9.4/−9.1 kcal/mol); sappanchalcone generated favorable cross-platform predictions at SRC (−6.9 to −8.5 kcal/mol). The MAPK1/3, SRC, and AKT1 nodes converge on MAPK activation, SRC-driven proliferation, and PI3K/AKT/mTOR survival signalling [21,22,23,24,25], and these pathways have been independently associated with HNC prognosis and field-cancerization biomarkers [24]. The exploratory docking signature reported here is therefore consistent with the direction of established mechanistic models, but it should be interpreted strictly as a structural-compatibility hypothesis: docking ΔG values are comparative predictions, not validated affinities [18,19,20]. ADMET and drug-likeness filtering (Lipinski and Veber compliance, GI absorption, bioavailability score 0.55) supports further experimental evaluation of brazilin and sappanchalcone but does not replace pharmacokinetic confirmation.

## Figures and Tables

**Table 1:** Cytotoxic, antioxidant, and anti-inflammatory activities and total flavonoid content of Thai ethnobotanical plants derived from Kampramong’s anticancer remedy (*n* = 3; mean ± SEM).

**Plant Species**	**Cytotoxic Activity** **(IC_50_ µg/mL)**								**NO** **(** **IC_50_ µg** **/** **mL** **)**	**DPPH** **(EC_50_ µg/mL)**	**TFC** **(mgQE/g)**
	**HK1**	**KB**	**HONE** **-** **1**	**NPC38**	**NPC43**	**HEp2**	**NP460**	**HaCaT**			
*A. ebracteatus*	19.59 ± 1.01	>100	19.09 ± 1.05	18.02 ± 1.08	20.00 ± 2.25	>100	98.03 ± 2.25	80.95 ± 0.94	5.90 ± 1.55	56.00 ± 1.01	151.32 ± 1.05
*A. paniculata*	38.40 ± 0.86	>100	38.40 ± 0.86	25.09 ± 1.99	30.00 ± 0.15	>100	>100	>100	30.82 ± 1.72	13.42 ± 1.65	101.35 ± 1.90
*A. sinensis*	>100	34.33 ± 0.17	35.80 ± 0.86	>100	>100	40.71 ± 0.02	>100	>100	10.52 ± 2.31	>100	10.15 ± 0.95
*A. vulgaris*	>100	>100	>100	>100	>100	>100	>100	27.30 ± 3.71	28.09 ± 1.25	73.65 ± 0.76	7.96 ± 1.20
*B. montanum*	>100	>100	>100	5.09 ± 1.08	4.41 ± 1.59	>100	>100	>100	6.06 ± 0.49	26.22 ± 0.88	48.66 ± 0.85
*B. strychnifolia*	>100	>100	>100	>100	>100	>100	>100	>100	>100	28.11 ± 0.46	29.32 ± 1.35
*B. alnoides*	39.39 ± 1.54	>100	39.39 ± 1.54	>100	>100	>100	>100	>100	40.09 ± 1.15	15.24 ± 1.16	56.55 ± 1.02
*C. sappan*	**7.22 ± 0.19**	**5.85 ± 0.21**	**7.22 ± 0.19**	**10.02 ± 1.03**	**13.60 ± 0.22**	**4.71 ± 1.41**	>100	**96.39 ± 0.02**	**9.35 ± 0.78**	**5.75 ± 0.86**	180.33 ± 0.95
*C. carandas*	>100	>100	>100	>100	>100	>100	>100	>100	>100	10.29 ± 1.98	19.35 ± 1.15
*C. garrettiana*	35.28 ± 1.09	29.41 ± 0.97	35.28 ± 1.09	5.92 ± 1.59	8.56 ± 0.31	30.16 ± 0.16	>100	99.22 ± 0.63	40.05 ± 5.17	4.06 ± 0.63	112.35 ± 1.85
*C. flavescens*	>100	>100	>100	>100	>100	>100	>100	>100	>100	>100	13.23 ± 1.00
*C. fenestratum*	**9.71 ± 1.02**	**5.96 ± 0.29**	**9.71 ± 1.02**	**8.04 ± 1.18**	**10.02 ± 1.53**	**6.31 ± 1.32**	>100	>100	**10.84 ± 1.28**	**19.12 ± 0.95**	68.95 ± 1.40
*C. oblongifolius*	20.07 ± 1.56	24.86 ± 1.13	20.07 ± 1.56	4.89 ± 1.05	3.54 ± 0.20	27.72 ± 0.12	95.93 ± 1.85	82.04 ± 3.15	10.95 ± 4.78	86.21 ± 1.69	115.34 ± 0.55
*F. foveolata*	>100	>100	>100	>100	>100	>100	>100	>100	>100	43.77 ± 2.18	4.18 ± 1.50
*H. perforata*	35.03 ± 1.19	40.92 ± 1.16	35.03 ± 1.19	25.08 ± 1.56	30.13 ± 1.45	38.62 ± 0.29	>100	>100	55.95 ± 5.21	16.91 ± 0.73	110.67 ± 0.95
*H. formicarum*	>100	>100	32.44 ± 0.86	>100	>100	40.42 ± 1.02	>100	>100	20.05 ± 1.65	3.47 ± 0.23	5.99 ± 1.55
*L. cubeba*	>100	>100	>100	>100	>100	>100	>100	>100	>100	19.48 ± 3.96	45.69 ± 1.35
*O. integerrima*	>100	>100	>100	>100	>100	>100	>100	>100	52.71 ± 1.55	19.48 ± 3.96	45.69 ± 1.35
*O. grandiflorus*	>100	36.53 ± 0.86	5.47 ± 1.13	>100	>100	>100	>100	>100	10.00 ± 0.15	7.59 ± 0.26	110.66 ± 1.95
*P. amarus*	5.07 ± 1.11	>100	5.07 ± 1.11	25.98 ± 1.02	22.18 ± 1.10	>100	92.03 ± 1.25	37.17 ± 5.65	10.48 ± 1.34	5.36 ± 1.56	159.69 ± 1.05
*P. reticulatus*	>100	>100	>100	>100	>100	>100	>100	>100	>100	8.83 ± 0.67	19.53 ± 1.35
*P. cerasoides*	29.89 ± 0.12	>100	37.32 ± 1.65	25.23 ± 0.19	37.89 ± 0.12	>100	>100	>100	25.49 ± 2.52	8.39 ± 0.09	125.69 ± 1.05
*R. nasutus*	80.79 ± 3.82	>100	30.66 ± 1.29	35.02 ± 1.16	54.42 ± 2.02	>100	>100	>100	>100	57.86 ± 0.68	39.09 ± 1.05
*S. chinensis*	>100	>100	>100	>100	>100	>100	>100	>100	>100	3.70 ± 0.17	49.85 ± 1.00
*S. corbularia*	>100	>100	>100	>100	>100	>100	>100	>100	83.90 ± 1.18	50.31 ± 0.91	89.35 ± 0.85
*S. grabra*	>100	60.00 ± 0.12	45.65 ± 1.32	>100	>100	>100	>100	>100	40.00 ± 0.41	3.12 ± 0.11	85.33 ± 0.95
*S. multiflora*	38.58 ± 1.28	>100	38.58 ± 1.28	>100	>100	>100	>100	>100	55.83 ± 0.65	>100	69.25 ± 1.05
*T. grandis*	**18.95 ± 0.21**	**18.95 ± 1.56**	**11.84 ± 0.24**	**15.02 ± 1.47**	**18.88 ± 1.13**	**20.00 ± 0.12**	>100	93.18 ± 2.20	**11.15 ± 3.58**	**13.23 ± 0.62**	150.14 ± 1.85
*T. triandra*	39.51 ± 0.14	>100	39.51 ± 0.14	22.26 ± 1.00	30.18 ± 1.15	>100	>100	>100	55.65 ± 5.34	15.38 ± 0.25	140.08 ± 1.25
*T. baenzigeri*	>100	>100	>100	>100	>100	>100	>100	>100	>100	14.18 ± 0.53	89.19 ± 0.05
*T. crispa*	>100	>100	>100	>100	>100	>100	>100	>100	>100	22.12 ± 1.31	53.23 ± 0.95
**Positive** **Control**	**Cytotoxic Activity** **(IC_50_ µg/mL)**								**NO** **(IC_50_ µg/mL)**	**DPPH** **(EC_50_ µg/mL)**	**TFC** **(mgQE/g)**
	**HK1**	**KB**	**HONE-1**	**NPC38**	**NPC43**	**HEp2**	**NP460**	**HaCaT**			
Cisplatin	25.39 ± 0.26	26.89 ± 0.16	10.73 ± 0.12	15.95 ± 0.58	7.05 ± 0.11	15.05 ± 0.11	96.92 ± 0.08	80.85 ± 3.15	-	-	-
(+/−) Brazilin	>100	35.50 ± 1.02	48.80 ± 1.12	20.50 ± 1.55	>100	30.05 ± 1.88	>100	>100	-	-	-
Prednisolone	-	-	-	-	-	-	-	-	1.31 ± 0.04	-	-
Ascorbic acid	-	-	-	-	-	-	-	-	-	4.50 ± 0.15	-

Note: Data are presented as mean ± SEM of three independent experiments (*n* = 3). NCI criteria: NCI criteria indicated three types of cytotoxic activity. A: active (IC_50_ value was below 20 µg/mL), MA: moderately active (IC_50_ value was between 20 and 100 µg/mL), and IA: inactive (IC_50_ value was above 100 µg/mL) [11]. Cisplatin IC_50_ values for all six cell lines (µg/mL ± SEM) and prednisolone IC_50_ for the anti inflammatory reference. Bold values indicate key experimental results of particular biological importance to facilitate clear identification and comparison of the most relevant findings.

**Table 2:** Component loadings, eigenvalues, % variance and communalities for the phytochemistry + bioactivity matrix of Kampramong’s anticancer remedy plant ingredients (*n* = 31 plants × 9 variables; data auto-scaled before PCA).

Variable	PC1 (F1, Loading)	PC2 (F2, Loading)	Communality (h^2^)
Flavonoid (TFC, mg QE/g)	−0.94	+0.21	0.93
IC_50_ HK1 (µg/mL)	+0.88	−0.27	0.85
IC_50_ KB (µg/mL)	+0.85	+0.41	0.89
IC_50_ HONE-1 (µg/mL)	+0.82	+0.39	0.83
IC_50_ NPC38 (µg/mL)	+0.79	+0.21	0.67
IC_50_ HEp2 (µg/mL)	+0.77	+0.45	0.80
NO inhibition (IC_50_, µg/mL)	+0.74	−0.05	0.55
IC_50_ NPC43 (µg/mL)	+0.72	−0.34	0.64
DPPH scavenging (EC_50_, µg/mL)	+0.68	−0.61	0.84
Eigen value	5.27	1.13	–
% variance	57.70	14.22	–
Cumulative % variance	57.70	71.92	–

Note: PC1 and PC2 explained 57.70% and 14.22% of the variance, respectively. PCA of the auto-scaled nine-variable matrix retained two components, explaining 71.92% of total variance (PC1 = 57.70%, PC2 = 14.22%). PC1 separated high-flavonoid extracts from high-IC50 profiles, indicating that higher TFC was associated with stronger cytotoxicity. PC2 mainly reflected DPPH-related variation. The loading structure supported the clustering of C. sappan, C. fenestratum, and T. grandis as the most promising extracts.

**Table 3:** Top targeted proteins involved in upper-airway carcinogenesis.

Rank	Gene	Protein	PBD ID	Node Score
1	MAPK3	Mitogen-activated protein kinase 3	4QTA	46
2	SRC	Proto-oncogene tyrosine-protein kinase Src	2H8H	45
3	MAPK1	Mitogen-activated protein kinase 1	4H3Q	43
4	AKT1	RAC-alpha serine/threonine-protein kinase	3O96	41
5	GRB2	Growth factor receptor-bound protein 2	1GRI	40

**Table 4:** Drug-likeness properties of the major *Caesalpinia sappan* compounds selected for docking.

Compound	Structure	MW g/mol	HBA	HBD	MLogP	Lipinski	Veber	GI Absorption	Bioavailability Score
(+/−) Brazilin	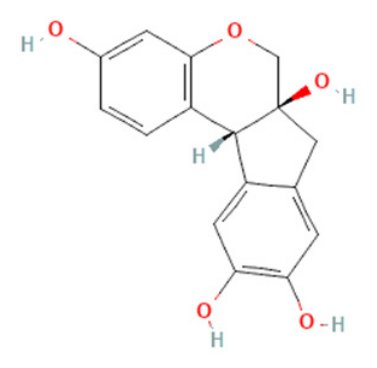	286.28	5	4	1.04	Yes	Yes	High	0.55
Sappanchalcone	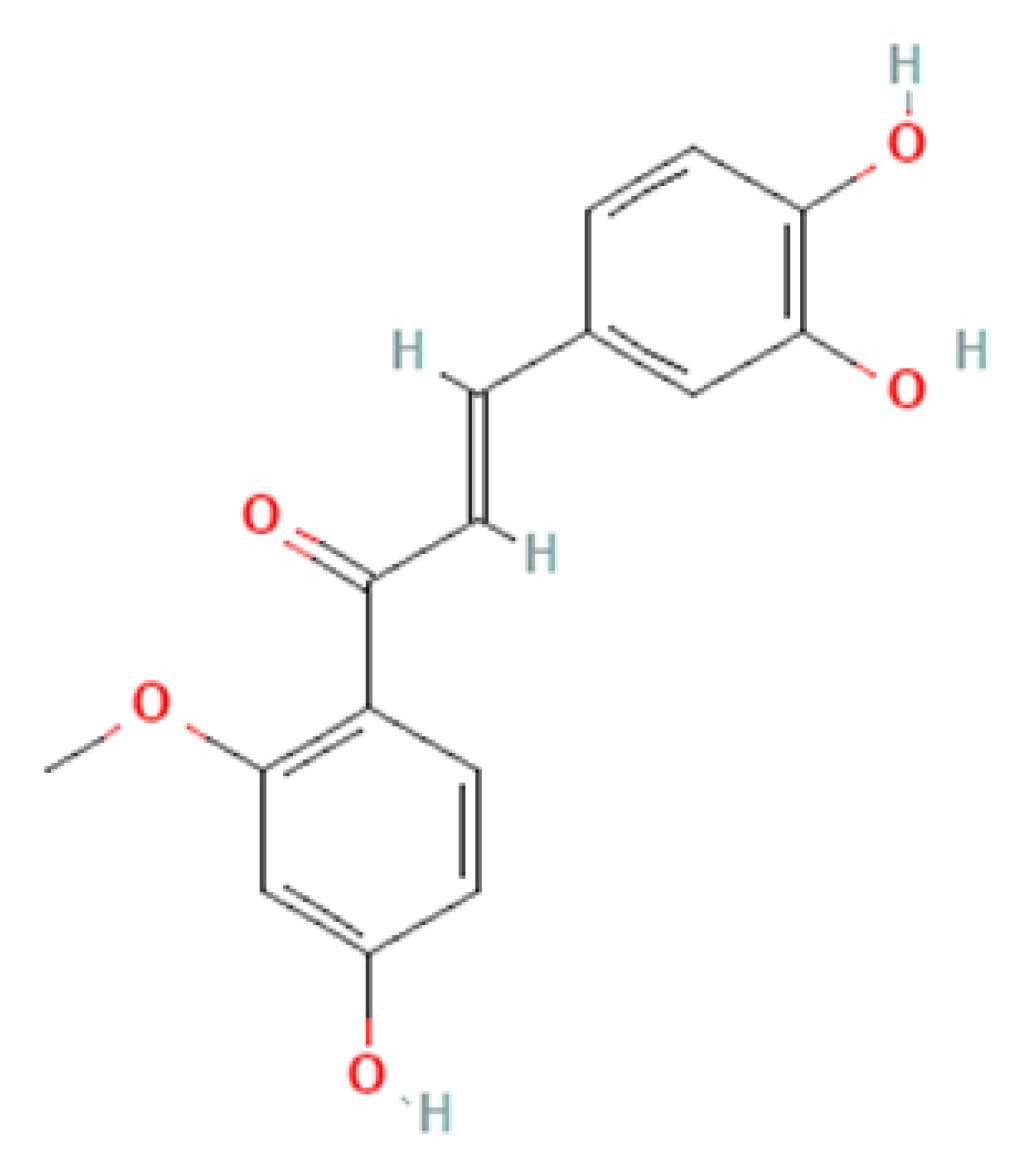	286.28	5	3	1.27	Yes	Yes	High	0.55

**Abbreviations:** MW, molecular weight (g/mol); HBA, hydrogen bond acceptors; HBD, hydrogen bond donors; MLogP, Moriguchi octanol/water partition coefficient; Lipinski, compliance with Lipinski’s Rule of Five; Veber, compliance with Veber’s Rule; GI absorption, predicted gastrointestinal absorption; Bioavailability score, predicted probability of oral bioavailability.

**Table 5:** Molecular docking ΔG (binding affinity, kcal/mol) of brazilin and sappanchalcone against the five core target proteins implicated in upper respiratory tract carcinogenesis; the MAPK1-MAPK3-SRC-AKT1-GRB2 target panel.

Protein	PDB ID	Resolution (Å)/R-Free	Highest Node-Score (String)	Brazilin ΔG (kcal/mol)	Sappanchalcone ΔG (kcal/mol)
MAPK1	(4H3Q)	1.85/0.249	43	**−8.6**	**−8.5**
MAPK3	(4QTA)	2.20/0.246	46	−7.6	−7.8
SRC kinase	(2H8H)	1.95/0.220	45	**−9.0**	−6.9
AKT1	(3O96)	1.65/0.214	41	**−8.1**	−7.5
GRB2 SH2	(1GRI)	1.80/0.225	40	**−9.6**	**−8.5**

Note: Bold values are below the −8.0 kcal/mol threshold. Scored by CB-Dock2 employs AutoDock Vina version 1.2.0 Vina. Higher negative values indicate stronger binding. Values ≤ −8.0 kcal/mol are considered tight-binding and represent promising ligand-protein pairs for downstream mechanistic study.

**Table 6:** Docking summary of C. sappan-derived phytochemicals against the MAPK1–MAPK3–SRC–AKT1–GRB2 panel.

Target	Compound	Binding Pocket	Key Interactions	CB-Dock_2_ ΔG (kcal/mol)	SeamDock ΔG (kcal/mol)	Estimated Ki (µM)	RMSD (Å)	Pose Validation Notes
MAPK1 (4H3Q)	3-Deoxysappanchalcone	ATP-binding cleft	π–π Tyr36; H-bond Asp167; hydrophobic packing	−8.0	−7.8	1.62 ± 0.04	1.42	Re-docked vs. native ligand; cross-checked with 4QTA/7OTE
MAPK1 (4H3Q)	(±)-Brazilin	Adjacent hydrophobic sub-pocket	H-bond Lys52; π–π Trp187; polar contacts Asp149/Asn152	−8.7	−8.5	0.49 ± 0.02	1.28	Vina single-pose ΔG = −8.6 ± 0.2; cross-platform consistency within 1 SD
MAPK3 (4QTA)	(±)-Brazilin	ATP-binding cleft	H-bond Met108; π–π Phe166; hydrophobic Val38/Ala50/Leu154	−9.4	−9.1	0.17 ± 0.01	1.16	Cross-platform consistency check: A loop-closed conformation

Note: Docking scores are comparative predictions. Estimated Ki values were calculated from mean ΔG using ΔG = RT ln Ki at T = 298.15 K. RMSD < 2.0 Å was used to support pose reproducibility, but functional validation is required [11,16,17,18].

**Table 7:** NO inhibition, DPPH antioxidant activity, and total flavonoid content of selected extracts.

Plant Species	NO IC_50_ (µg/mL)	DPPH EC_50_ (µg/mL)	TFC (mgQE/g)
*C. sappan*	9.35 ± 0.78	5.75 ± 0.86	180.33 ± 0.95
*C. fenestratum*	10.84 ± 1.28	19.12 ± 0.95	68.95 ± 1.40
*T. grandis*	11.15 ± 3.58	13.23 ± 0.62	150.14 ± 1.85
Prednisolone (positive NO control)	1.31 ± 0.04	—	—
Ascorbic acid (positive DPPH control)	—	4.50 ± 0.15	—

**Note:** Data are presented as mean ± SEM (*n* = 3). IC_50_ indicates the concentration required to inhibit NO production by 50%, whereas EC_50_ indicates the concentration required to scavenge 50% of DPPH radicals. TFC is expressed as mg quercetin equivalents per gram of extract (mgQE/g). Prednisolone and ascorbic acid served as positive controls for NO inhibition and DPPH antioxidant activity, respectively. “—” denotes not tested/not applicable.

**Table 8:** Crosswalk between *C. sappan* cytotoxicity and predicted docking scores.

Cell Line	*C. sappan* IC50 (µg/mL)	Cisplatin IC50 (µg/mL)	*C. sappan* SI (vs. HaCaT)	Cisplatin SI (vs. HaCaT)	Closest Docking ΔG Anchor (kcal/mol)
HK1	7.22 ± 0.19	25.39 ± 0.26	13.35	3.18	(±)-Brazilin@MAPK3 (−9.4/−9.1)
KB	5.85 ± 0.21	26.89 ± 0.16	16.48	3.01	(±)-Brazilin@MAPK1 (−8.7/−8.5)
HONE-1	7.22 ± 0.19	10.73 ± 0.12	13.35	7.54	(±)-Brazilin@MAPK3 (−9.4/−9.1)
NPC38	10.02 ± 1.03	15.95 ± 0.58	9.62	5.07	(±)-Brazilin@MAPK1 (−8.7/−8.5)
NPC43	13.60 ± 0.22	7.05 ± 0.11	7.09	11.47	(±)-Brazilin@MAPK3 (−9.4/−9.1)
HEp2	4.71 ± 1.41	15.05 ± 0.11	20.47	5.37	Sappanone A@AKT1 allosteric (−8.1/−7.9)

**Note:** Data are presented as mean ± SEM. IC_50_ indicates the concentration required to reduce cell viability by 50%. SI (selectivity index) was calculated as the IC_50_ value in non-cancerous HaCaT cells divided by the IC_50_ value in each cancer cell line. Higher SI values indicate greater selectivity toward cancer cells relative to HaCaT cells. Cisplatin was used as the positive cytotoxic control. Docking ΔG values represent predicted binding free energies; more negative values indicate stronger predicted ligand–target binding. Docking results are computational predictions and should be interpreted as mechanistic hypotheses requiring experimental validation.

## Data Availability

The data presented are available on request from the corresponding author.

## Supplementary Materials

The following supporting information can be downloaded at: https://www.mdpi.com/article/10.3390/ijms27167239/s1.

## Abbreviations

The following abbreviations are used in this manuscript:

ADME	Absorption, distribution, metabolism, and excretion
ATCC	American Type Culture Collection
Bioavailability score	Predicted probability of oral bioavailability
CI	Confidence interval
CLS	Cell Lines Service GmbH
DKSFM	Defined keratinocyte serum-free medium
DMEM	Dulbecco’s Modified Eagle Medium
DMSO	Dimethyl sulfoxide
DPPH	2,2-Diphenyl-1-picrylhydrazyl radical
EC_50_	Half-maximal effective concentration
FBS	Fetal bovine serum
FDR	False discovery rate
GI absorption	Predicted gastrointestinal absorption
HBA	Hydrogen bond acceptors
HBD	Hydrogen bond donors
HCA	Hierarchical cluster analysis
HNC	Head and neck cancer
HNSCC	Head and neck squamous cell carcinoma
HR	Hazard ratio
IC_50_	Half-maximal inhibitory concentration
KM	Kaplan–Meier
Lipinski	Compliance with Lipinski’s Rule of Five
LPS	Lipopolysaccharide
MEM	Minimum Essential Medium
MLogP	Moriguchi octanol/water partition coefficient
MTT	3--2,5-diphenyltetrazolium bromide
MW	Molecular weight
NCS	Newborn calf serum
NO	Nitric oxide
NPC	Nasopharyngeal carcinoma
OD	Optical density
PBS	Phosphate-buffered saline
PCA	Principal component analysis
PDB	Protein Data Bank
PDBQT	Protein Data Bank, partial charges and atom types
PPI	Protein–protein interaction
Q1/Q3	First/third quartiles
RMSD	Root-mean-square deviation
RPMI-1640	Roswell Park Memorial Institute Medium 1640
SD	Standard deviation
SEM	Standard error of the mean
SI	Selectivity index
SRB	Sulforhodamine B
STRING	Search Tool for the Retrieval of Interacting Genes/Proteins
TCA	Trichloroacetic acid
TFC	Total flavonoid content
TTM	Traditional Thai medicine
URT	Upper respiratory tract
Veber	Compliance with Veber’s rules
