# Supplementary material for: Cytotoxic and Anti-Inflammatory Evaluation of Thai Medicinal Plants from Kampramong’s Anticancer Remedy Against Upper Respiratory Tract Malignancies
Source: Int J Mol Sci. 2026 Aug 13;27(16):7239. doi: 10.3390/ijms27167239 (PMC13513281; doi:10.3390/ijms27167239)
Supplement: Supplementary file 1 [file ijms-27-07239-s001.zip › ijms-4456195-supplementary.pdf]

Table S1. The botanical details, biological activity, and traditional use of Thai ethnobotany ingredients in Kampramong's anticancer remedy.

**Plant species**	**Family**	**Part used**	**Voucher specimen number**	**%Yield**	**Traditional use***
Acanthus ebracteatus Vahl	Acanthaceae	All parts	SKP 001 01 05 01	5.60	Chronic ulcer healing
Andrographis paniculata (Burm.f.) Wall.ex Nees	Acanthaceae	All parts	SKP 001 01 16 01	3.62	Antipyretic
Angelica sinensis (Oliv.) Diels	Umbelliferae	Root	SKP 199 01 19 01	7.24	Antipyretic and cough relief
Artemisia vulgaris Linn.	Compositae	All parts	SKP 051 01 22 01	3.64	Antipyretic and cough relief
Baliospermum montanum (Willd.) Muell. Arg	Euphorbiaceae	Root	SKP 071 02 13 01	1.95	Antipyretic and anemia treatment
Bauhinia strychnifolia Craib.	Leguminosae	All parts	SKP 098 02 19 01	15.54	Nourishes the blood and heart and body tonic
Betula alnoides Buch-Ham.ex G.Don	Betulaceae	Bark	SKP 024 02 01 01	1.37	Nourishes the blood, antipyretic, and body tonic
Caesalpinia sappan L.	Caesalpinioideae	Stem	SKP 072 03 19 01	4.93	Nourishes the blood
Carissa carandas Linn.	Apocynaceae	Root	SKP 072 03 03 01	1.50	Appetizer, anti-sore throat, and anti-diarrhea
Cassia garrettiana (Craib) Irwin & Barneby	Caesalpinioideae	Heartwood	SKP 034 03 07 01	2.03	Nourishes the blood
Coptosapelta flavescens Korth.	Rubiaceae	Stem	SKP 165 03 06 01	5.42	Pain relief
Coscinium fenestratum (Goetgh.) Colebr.	Menispermaceae	Stem	SKP 114 03 06 01	4.52	Anti-pyretic, treating jaundice, and anti-flatulence
Croton oblongifolius Roxb.	Euphorbiaceae	Stem	SKP 071 03 15 01	2.52	Nourishes the blood
Ficus foveolata Wall.	Moraceae	Stem	SKP 117 06 06 01	2.05	Nourishes the blood and body tonic
Harrisonia perforata (Blanco) Merr.	Simaroubaceae	Root	SKP 178 08 16 01	1.68	Anti-pyretic and anti-diarrhea
Hydnophytum formicarum Jack	Rubiaceae	Rhizome	SKP 165 08 06 01	8.32	Anticancer and nourishes the heart
Litsea cubeba (Lour.)	Lauraceae	Stem	SKP 096 12 03 01	1.01	Anti-flatulence
Ochna integerrima (Lour.) Merr.	Ochnaceae	Stem	SKP 218 15 09 01	3.21	Nourishes the blood and body tonic
Orthosiphon grandiflorus Bold.	Lamiaceae	All parts	SKP 095 15 07 01	4.47	Diuretic
Phyllanthus amarus Schumach. & Thonn.	Euphorbiaceae	Aerial part	SKP 071 16 01 01	5.24	Antipyretic, nourishes the blood, and alleviates body pain
Phyllanthus reticulatus Poir.	Phyllanthaceae	Root	SKP 071 16 18 01	1.52	Antipyretic, treating abscess, and abdominal pain relief
Polyalthia cerasoides (Roxb.) Benth. ex Bedd. ST	Annonaceae	Bark	SKP 011 16 03 01	5.14	Pain relief and anti-tuberculosis

Rhinacanthus nasutus (L.) Kurz.	Acanthaceae	All part	SKP 001 18 14 01	1.28	Treatment of skin infection, eczema, and scabies
Salacia chinensis L.	Celastraceae	Bark	SKP 004 19 03 01	4.57	Antipyretic, pain relief, and treating hepatomegaly
Smilax corbularia Kunth	Smilacaceae	Rhizome	SKP 202 16 08 01	4.26	Improve the lymphatic system
Smilax grabra Roxb.	Smilacaceae	Rhizome	SKP 179 19 07 01	8.86	Improve the lymphatic system
Suregada multiflora (A. Juss) Baill.	Euphorbiaceae	Stem	SKP 071 19 13 01	1.91	Nourishes the blood, antipyretic, and anticancer properties
Tectona grandis L.f.	Labiatae	Stem	SKP 095 20 07 01	2.85	Antipyretic, nourishes the blood, and element of the body
Tiliacora triandra (Colebr.) Diels	Menispermaceae	Root	SKP 114 20 20 01	4.65	Antipyretic and anticancer properties
Tinospora baenzigeri Forman	Menispermaceae	Stem	SKP 114 20 02 01	2.22	Body tonic, appetizer, and anticancer properties
Tinospora crispa (L.) Miers ex Hook.f. & Thomson.	Menispermaceae	Stem	SKP 114 20 03 01	3.08	Antipyretic, body tonic, appetizer, and treating jaundice
